# Systematic review and meta-analysis on the mental health of emergency and urgent call-handlers and dispatchers

**DOI:** 10.1093/occmed/kqae104

**Published:** 2024-11-04

**Authors:** C Osório, S Talwar, S A M Stevelink, H K Sihre, D Lamb, J Billings

**Affiliations:** Division of Psychiatry, University College London (UCL), London W1T 7NF, UK; South London and Maudsley NHS Foundation Trust, Talking Therapies Southwark, Guy’s Hospital, London SE1 3SS, UK; Division of Psychiatry, University College London (UCL), London W1T 7NF, UK; Department of Psychological Medicine, Institute of Psychiatry, Psychology and Neuroscience (IoPPN), Weston Education Centre, King’s College London, London SE5 9RJ, UK; Department of Primary Care and Population Health, University College London (UCL), London WC1E 6BT, UK; NIHR Applied Research Collaboration North Thames, London WC1E 6BT, UK; Department of Primary Care and Population Health, University College London (UCL), London WC1E 6BT, UK; NIHR Applied Research Collaboration North Thames, London WC1E 6BT, UK; Division of Psychiatry, University College London (UCL), London W1T 7NF, UK

## Abstract

**Background:**

Call-handlers and dispatchers (CHDs) working in specialized emergency and urgent communication centres are essential in supporting public safety and health. Evidence suggests that these professionals are at increased risk of mental health conditions, including post-traumatic stress disorder (PTSD), depression, anxiety and alcohol abuse among other conditions; however, reliable prevalence estimates remain undetermined.

**Aims:**

We provide the estimated pooled prevalence for PTSD, depression, anxiety and hazardous drinking among emergency and urgent CHDs globally.

**Methods:**

We searched 10 electronic databases, grey literature and the Annals of Emergency Dispatch and Response. Eligible studies reported original quantitative data and used validated self-reported measures on the prevalence of mental health conditions of interest (i.e. PTSD, depression, anxiety and alcohol use) within CHD professionals. Three reviewers independently screened results for eligibility. Prevalence estimates were pooled using random-effects meta-analyses.

**Results:**

In total, 16 857 references were retrieved. From these, 183 full-text articles were assessed for eligibility, of which 37 articles were included in this systematic review, and 13 articles provided necessary quantitative information for meta-analyses. In total, 7759 CHDs were considered across nine different countries. The overall pooled prevalence for PTSD was 17.8% (95% confidence interval [CI] 12.4−24.0%), depression was 28.2% (95% CI 20.7–36.2%), anxiety was 17.2% (95% CI 6.6–31.5%) and hazardous drinking was 17.8% (95% CI 6.9–32.2%).

**Conclusions:**

These findings indicate CHDs are at significant risk of mental health problems. Further quantitative and qualitative research is warranted to help understand the psychological risks of working as a CHD and guide appropriate psychological support.

## Teaser

Call-handlers and dispatchers (CHDs) provide critical telephone-based support, and research suggests an increased risk of mental health problems. There are limited meta-analyses available on post-traumatic stress disorder (PTSD), depression, anxiety and alcohol use in CHDs. Our meta-analysis shows a pooled prevalence of PTSD of 17.8%, depression of 28.2%, anxiety of 17.2% and hazardous drinking of 17.8%. These findings suggest that almost 3 in 10 CHDs report depression, while 1 in 5 report PTSD, anxiety or hazardous drinking.

## Introduction

Call-handlers and dispatchers (CHDs) are the first point of access in an emergency or urgent situation, and they provide critical telephone-based advice and support 24 hours a day, 7 days a week and 365 days a year [1,2]. CHD professionals operating within the ambulance, police and fire service communication centres often work under high pressure and deal with life-threatening scenarios, including members of the public reporting heart attacks, drug overdoses, suicidal ideation, homicide, fires and other life-threatening emergencies [3]. CHDs must assess and communicate complex information, answer questions, identify the emergency/urgency, establish where the incident is happening, the threat level and appropriate emergency/urgent response, and, in some scenarios, coordinate a combined emergency response [1]. The above actions must be executed rapidly with the use of manualized and/or computer-aided approaches while engaging with distressed members of the public or emergency services. Once an emergency/urgent call is managed, this process is repeated throughout a working shift [1,2,4].

CHD professionals have been a focus of mental health research due to the high-risk nature of their profession. Empirical evidence concerning these professionals has shown that repeated exposure to stress and trauma (e.g. frequent distressing and graphic calls, long shifts and unsupportive work environments) was associated with increased mental health problems, particularly post-traumatic stress disorder (PTSD), depression, anxiety, alcohol abuse and sleep difficulties [1,2,4].

Currently, there is limited epidemiological meta-analytic research on the mental health of CHDs. In contrast, frontline first responders (ambulance and police professionals) have recently been the focus of two meta-analyses [5,6]. These meta-analyses showed a pooled prevalence estimate for ambulance personnel and police personnel respectively of PTSD of 11.0% (95% confidence interval [CI] 7.0–14.0%) and 14.2% (95% CI 10.3–18.7%), depression of 14.6% (95% CI 10.9–18.6%) and 15.0% (95% CI 10.0–20.0%), anxiety of 9.6% (95% CI 6.7–12.9%) and 15.0% (95% CI 8.0–22.0%) and hazardous drinking of 25.7% (95% CI 19.6–32.4%) [5,6]. These findings indicate a relatively high prevalence of mental health difficulties in frontline first responders, which may suggest similar risks for CHDs. However, even within frontline first responders, there is considerable variation in prevalence estimates, which makes generalizing findings to CHDs problematic, given that CHDs operate in a different work setting.

This systematic review aimed to review and summarize data from the literature on mental health and well-being and complete a meta-analysis on the prevalence of PTSD, depression, anxiety and hazardous drinking among CHDs worldwide working in specialized emergency and urgent communication centres.

## Methods

This systematic review and meta-analysis were developed in accordance with the Preferred Reporting Items for Systematic Reviews and Meta-Analyses (PRISMA) Guidelines [7]. The review protocol was prospectively registered online on the PROSPERO Database (Registration number: CRD42022312093) [8].

We searched 10 electronic databases (Medline [via Ovid], PubMed, APA PsycARTICLES [via Ovid], APA PsycEXTRA [via Ovid], APA PsycINFO [via Ovid], EMBASE [via Ovid], Web of Science, CINAHL Plus [via EBSCOhost] and Cochrane Library for articles published from inception to 7 January 2024 in English, French, Spanish, Italian and Portuguese). A further manual search was conducted using Google Scholar in English and Portuguese. We independently searched the content of a leading academic journal for research with emergency responders and dispatchers, *Annals of Emergency Dispatch and Response (AEDR)*. We independently searched this journal, as PubMed and Medline databases are not indexed, and relevant scientific papers would have been missed.

To identify relevant academic literature concerning common mental disorders in CHDs working in specialized emergency and urgent communication centres, we compiled a comprehensive list of search terms in consultation with a specialist librarian. We used Medical Subject Headings (MeSH-specific to Medline), subject headings, index terms and text word searching, in combination with Boolean Operators ‘AND’ and ‘OR’ to link specific search terms from (i) CHDs (including emergency call-handlers, emergency dispatchers, crisis calls), and (ii) mental health and well-being (including PTSD, anxiety, resilience) together, to identify relevant scientific articles in each electronic database. We manually searched the reference list of excluded systematic reviews with CHDs for further papers [1,2]. We also did backwards and forward citation searching of included articles. We used Google Scholar to search for grey literature. The Google Scholar search strategy included combining keywords in English language such as ‘emergency’, ‘urgent’, ‘telecommunicators’, ‘mental health’ and ‘wellbeing’ and in the Portuguese language, such as ‘emergência’, ‘urgência’, ‘operadores de telecomunicação’, ‘saúde mental’ and ‘bem-estar’. Then, we searched the first 10 webpages in both English and Portuguese. Please see Table 1 (available as Supplementary data at *Occupational Medicine* online) for the full search strategy.

Study eligibility was determined by the Participants, Intervention, Comparator, Outcome Measures and Study design (PICOS) criteria [9]. These included all current and former professionals working as CHDs (e.g. ambulance, police, fire service, search and rescue) in an emergency and/or urgent specialized communication centre. Studies that only included frontline emergency responders (e.g. paramedics, police officers, firefighters) who physically respond to the emergency or urgency, or staff working on voluntary helplines (e.g. Samaritans, suicide prevention lifeline) or staff working in customer call centres (e.g. retail, financial services) were excluded from this review. We included original studies that reported on at least one of the following mental health outcomes; PTSD, depression, anxiety, burnout, alcohol abuse, stress and resilience, using validated standardized self-reported measures (e.g. questionnaires, scales), clinician-administered measures (e.g. Structured Clinical Interview for the Diagnostic Statistical Manual [DSM] of Mental Disorders [SCID] or the Clinician-Administered Scale for DSM-5 [CAPS-5]), diagnosis in professional settings and cut-off scores. We considered peer-reviewed articles with various research designs (e.g. cross-sectional studies, case–control studies, cohort studies). We excluded review articles (e.g. systematic reviews, meta-analyses), research protocols, non-peer-reviewed articles (e.g. masters and doctoral theses, government reports), case studies and qualitative studies of participants’ experiences.

When different articles included data on the same sample, we extracted data from all articles but prioritized the highest National Institute of Health (NIH) Quality-rated study or the most relevant article (e.g. the article that best described the mental health outcomes of CHDs) [10]. If these articles were rated the same score, we considered the one with the most relevant information or the most recently published.

All retrieved titles and abstracts were uploaded to EndNote and de-duplicated [11]. Subsequently, all data were uploaded to Rayyan Software (systematic review software) for study selection, reporting and further de-duplication [12]. Identified articles were independently screened by two researchers (researcher one [C.O.] and researcher two [S.T.]). Researcher one screened all titles and abstracts, while researcher two screened 7.5% (*n* = 866). A full-text screen of articles against the inclusion criteria was completed to determine eligibility. Other retrieved relevant articles identified from backward and forward citations searching were also examined in a full-text review. Researcher one screened all full-text articles, while researcher two screened 34.0% (*n* = 62). Any disagreement between researcher one and researcher two was resolved via a verbal or written agreement with the third independent researcher (H.S.).

The methodological quality and risk of bias of each article included in the systematic review and meta-analysis (*n* = 37) were independently assessed by researcher one and researcher two. Researcher one assessed the quality of all articles, while researcher two assessed 91.9% (*n* = 34). Three articles only had one reviewer due to the researcher two not being fully proficient in French and Spanish languages.

To assess case–control study designs, we used the original version of the NIH Quality Assessment tool for case–control studies (12 items). To assess cohort or cross-sectional study designs, we used the original version of the NIH Quality Assessment tool for observational cohort and cross-sectional studies (14 items) [10]. Each item in these two NIH tools assists reviewers in identifying potential flaws in study methodology, including sources of bias (e.g. patient selection, performance, attrition), study power and control for confounding variables, among others. Reviewers choose between the option ‘yes’, ‘no’, ‘cannot determine’, ‘not applicable’ and ‘not reported’ options. The higher presence of ‘no’, ‘cannot determine’ or ‘not reported’ in the NIH tool highlights the risk of potential methodological flaws in the reviewed study; however, higher weight is given to the ‘no’ response. Lastly, a final overall quality rating of ‘good’, ‘fair’ or ‘poor’ is then given to each study, considering their potential flaws in implementation and design. A quality rating of ‘good’ highlights that the study is low in risk of bias, and its results are deemed valid. A quality rating of ‘fair’ suggests the study is susceptible to some bias, but this is insufficient for the results to be considered invalid. Finally, a quality rating of ‘poor’ indicates a substantial risk of bias and significant flaws in the particular study [10]. All rating discrepancies were internally resolved between the two researchers. It was part of the protocol that disagreements would be decided via the third researcher, but this was not required.

The data extraction of included studies (*n* = 37) was performed by researcher one, using a custom-designed Excel spreadsheet. Researcher two independently assessed a random selection of the full-text articles (18.9%, *n* = 7) for information accuracy. Information was extracted on study characteristics (e.g. country of study, study design, sampling method), participants’ characteristics (e.g. sex, age, education, work role, work experience), study objectives (e.g. main and secondary objectives), recruitment strategy (e.g. inclusion/exclusion criteria, recruitment and procedure, initial sample, control group, final sample, dropout, representative of the population) and study outcomes (e.g. screening measures and cut-offs, main findings and summary and conclusions). Where articles were missing information critical for the systematic review and meta-analysis (*n*  = 4), the authors were contacted by email to provide this information with a deadline of 2 months. Two authors replied to the email—one of them provided further relevant information for use in this systematic review, and the other author mentioned that information about CHDs was not identifiable in their database, so the article was excluded. The remaining two authors did not reply to the email within the established timeframe, and the articles in question were excluded.

All studies included in the meta-analysis provided numerical data about the prevalence estimates (e.g. PTSD, depression, anxiety and hazardous drinking), and/or suitable subgroups and meta-regression variables (e.g. year of publication, sample size, mean age, proportion of females, type of questionnaires). When an included study used two different cut-off scores to define caseness (e.g. IES-R cut-off score >22 and >33) we opted for the most commonly used cut-off score in our review (e.g. IES-R cut-off score >33). When a study provided both a cut-off score and a diagnosis via clustering score to define a positive case, we only considered the cut-off score measure. This method allowed an estimation of a more uniform pooled prevalence estimate in the meta-analysis. We used the mathematical ‘simple rule of three’ method to solve proportional problems when an article in our meta-analysis provided the total number of cases and the prevalence estimate but did not provide the number of positive cases. Without identifying the number of positive cases, we could not perform a meta-analysis [13]. All data were extracted and imported to Stata Version 18 for statistical analyses [14].

The overall pooled prevalence estimates for PTSD, depression, anxiety and hazardous drinking were calculated via the *metaprop* Stata command. Since we expected high levels of heterogeneity, we employed a conservative approach to calculating the pooled prevalence estimate and used the random-effects meta-analysis with the Restricted Maximum Likelihood variance estimation method [14–16]. The Freeman–Tukey Double Arcsine Transformation was used to stabilize the variances and adjust the data to a more normal distribution [16]. We used pooled prevalence estimates to define mean prevalence estimates and 95% CI.

Between-study variability was calculated via the chi-squared (χ^2^) test (also known as Cochran’s *Q* test), the *I*-squared (*I*^2^) test and the tau-squared (χ^2^) test [16]. The χ^2^ test examined whether the individual studies’ outcomes were outside the estimated common effect, beyond what is expected by chance. A high χ^2^ test value indicates heterogeneity [17]. The *I*^2^ test informed the percentage of variance attributed to heterogeneity, instead of sampling error [17]. The range of *I*^2^ varies between 0% and 100%, and a percentage score of 50–90% represents ‘substantial heterogeneity’, and between 75% and 100% indicates ‘considerable heterogeneity’ [17]. The *I*^2^ test can be biased to both small and large meta-analyses, so, we also included theτ τ^2^ test [18]. The τ^2^ test provided an estimated variance of the true effect size, as its results are insensitive to the number of studies in the meta-analysis [18].

Sensitivity analyses were conducted to explore the impact of each individual study on PTSD, depression, anxiety and hazardous drinking prevalence estimates [17].

We ran separate meta-analyses for PTSD, depression, anxiety and hazardous drinking to study how the pooled prevalence estimate varied according to study characteristics (e.g. year of publication, continent), sample characteristics (e.g. age, proportion of females) and outcome characteristics (e.g. type of questionnaires used) [16].

Separate subgroup and meta-regression analyses were calculated via the *metareg* Stata command. These analyses investigated whether variables (e.g. study characteristics, sample characteristics and outcome characteristics) explained the between-study heterogeneity. To run these analyses, we converted PTSD, depression, anxiety and hazardous drinking prevalence estimates into log odd ratios (ORs) and then were transformed back, which provided an approximate estimation of the ORs. We employed random-effect models with the Knapp–Hartung variance estimator to ensure greater precision pooling of the prevalence estimation [16, 19].

Finally, publication bias was examined via the *metafunnel* and *metabias* Stata commands. Publication bias was assessed by visually examining the funnel plot for evidence of asymmetry and by running a linear regression to examine the association between the standard error and standardized effect estimate (Egger’s regression test). Publication bias was considered present if Egger’s regression test had a value of *P* ≤ 0.05 [6,20]. We conducted the funnel plots and Egger’s regression tests for PTSD, depression, anxiety and hazardous drinking.

## Results

**Figure 1.**
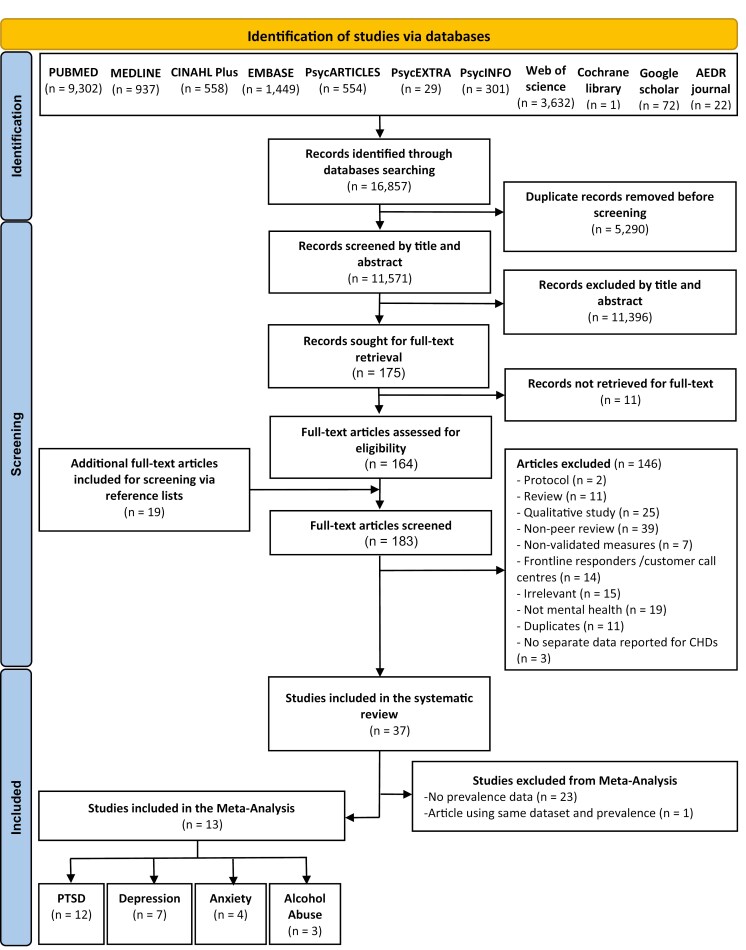
Results of the systematic literature review and meta-analysis.

The database searches yielded a total of 16 857 records. After the removal of 5290 duplicate records, a total of 11 571 articles were screened by title and abstract against the inclusion/exclusion criteria. A total of 11 396 records were excluded, leaving 183 which were then screened at full-text stage. In total, 37 articles were included in this systematic review and of those, 13 articles contained sufficient data to be included in the meta-analysis. We excluded organizational stress from the meta-analysis as we only found three prevalence studies, and this does not correspond to a psychiatric condition. Please see Figure 1 for the PRISMA flowchart of the screening and study selection process.

Seven articles received a quality rating of ‘good’ (18.9%), 29 articles a quality rating of ‘fair’ (78.4%) and one article a quality rating of ‘poor’ (2.7%). The majority of our studies received a quality rating of ‘fair’, and this was due to articles being unclear about the response rate, not controlling for previous traumatic experiences at work, or not adjusting for key potential confounders variables. Please see Table 2 (available as Supplementary data at *Occupational Medicine* online) for the full-quality assessment tables.

Of the 37 articles included, 31 were cross-sectional studies, 4 were case–control studies and 2 were cohort studies [3,21–56]. The included articles were published between 1988 and 2023, with most articles published since 2001 (35 out of 37 articles). All studies included in this review used self-reported measures as their method of diagnosis. In total, 7759 CHDs were considered across nine different countries, with most studies conducted in America (64.8%) and Europe (26.5%). The population were majority female (72.0%), of White ethnicity (78.4%) and with a median age of 38.5 years (age interquartile range [IQR] 34.1–41.6 years). The professionals were predominantly dispatchers (47.1%), working in 999/911 emergency response settings (89.7%) and employed by the medical services (34.8%). Please see Table 3 for the included study and sample characteristics.

**Table 3.** Included study and sample characteristics

Study characteristics	Number of studies (%)
**Year of publication** (studies = 34)^a^	
1980 to 1999	2 (5.9)
2000 to 2019	18 (52.9)
2020 to 2024	14 (41.2)
**Continent** (studies = 34)^a^	
Europe	9 (26.5)
Australia	1 (2.9)
Asia	1 (2.9)
North America	22 (64.8)
South America	1 (2.9)
**Sample size CHDs** (studies = 34)^a^	
<250	23 (67.6)
≥250	11 (32.4)
**Response rate** (studies = 34)^a^	
Not reported	15 (44.1)
<50%	6 (17.7)
≥50%	13 (38.2)
**Sample characteristics**	**Number of studies (%)**
**Age** (studies = 20)^b^	Median = 38.5 years (IQR = 7.5) IQR = 34.1–41.6 years
**Proportion of females** (studies = 30)	Median = 72.0% (IQR = 25.2) (Medium = 96 females) (10–690 females) Total = 4859
**Proportion of white ethnicity** (studies = 14)	Median = 78.4% (IQR = 25.9) (Medium = 150 white participants) (9–710 white ethnicity) Total = 3458 white ethnicity
**Years in service** (studies = 11)	Median = 11.9 years of experience (IQR = 8.9) IQR = 3.8–12.7 years of experience
**Work role** (studies = 34)	Total = 7759 CHDs
Call-handlers	8 (23.5)
Dispatchers	16 (47.1)
Call-handlers and dispatchers	8 (23.5)
Various roles (mixed roles)^c^	2 (5.9)
**Sector of employment** (studies = 23)	
Medical services	8 (34.8)
Police services	8 (34.8)
Search and rescue services	1 (4.3)
Various services (mixed services)^d^	6 (26.1)
**Type of response** (studies = 29)	
Emergency response (e.g. 999, 911)	26 (89.7)
Urgent response (e.g. 111, 101)	2 (6.9)
Emergency and urgent response	1 (3.4)
**Outcome characteristics** ^e^	**Number of studies (%)**
**Type of PTSD scale** (studies = 12)	
PCL questionnaire	7 (58.3)
IES questionnaire	3 (25.0)
PC-PTSD questionnaire	1 (8.3)
PDS questionnaire	1 (8.3)
**Type of depression scale** (studies = 7)	
BDI questionnaire	3 (42.9)
PHQ-9 questionnaire	3 (42.9)
PHQ-4 questionnaire	1 (14.3)
** Type of depression scale** (studies = 4)	
GAD-7 questionnaire	3 (75.0)
PHQ-4 questionnaire	1 (25.0)
** Type of alcohol scale** (studies = 3)	
AUDIT questionnaire	2 (66.7)
CAGE questionnaire	1 (33.3)

^a^Three studies were not included, as they contained the same sample.

^b^Studies that only included age as a numerical value. Studies with age as a categorical value were not included.

^c^Mixed roles—sample contained various professionals, including call-handlers and/or dispatchers but information is not provided.

^d^Mixed services—sample contained various services, including medical and police services.

^e^Outcome characteristics that were included in the meta-analysis.

Abbreviations: IQR—Interquartile Range; PCL—Post-Traumatic Stress Disorder Checklist; IES—Impact Event Scale; PC-PTSD—Primary Care Post-Traumatic Stress Disorder; PDS—Posttraumatic Stress Diagnostic Scale; BDI—Beck Depression Inventory; PHQ-9—Patient Health Questionnaire 9; PHQ-4—Patient Health Questionnaire 4; GAD-7—Generalised Anxiety Disorder 7; AUDIT—Alcohol Use Disorder Identification Test; CAGE—Cut, Annoyed, Guilty and Eye.

Of the 37 studies in this systematic review, 14 (37.8%) assessed four or more mental health and welfare outcomes, including probable mental health disorders, employment-related experiences, emotional awareness and psychological well-being. The most commonly examined mental health disorders were PTSD (*n* = 17), stress (*n* = 16), psychological distress (*n* = 10) and depression (*n* = 9), among others. Employment-related experiences included job satisfaction (*n* = 8), social support (*n* = 3) and coping styles (*n* = 3), among others. Emotional awareness included feelings/emotions (*n* = 8), mindfulness (*n* = 2) and self-efficacy (*n* = 2). Psychological well-being included post-traumatic growth (PTG) and/or resilience (*n* = 5), and well-being (*n* = 3). Please see Table 4 (available as Supplementary data at *Occupational Medicine* online) for a detailed summary of the included studies.

A total of 12 studies assessed the prevalence of PTSD and included, for example, the PTSD Checklist (PCL-C or PCL-5, *n* = 7), Impact Event Scale-Revised (IES-R, *n* = 3), Primary Care PTSD (PC-PTSD, *n* = 1) and the Post-Traumatic Stress Diagnostic Scale (PDS, *n* = 1). Seven studies assessed the prevalence of depression and included the Patient Health Questionnaire ([PHQ-9, *n* = 3] and PHQ-4, *n* = 1]) and the Beck Depression Inventory (BDI or BDI-II, *n* = 3). Four studies assessed the prevalence of anxiety via the Generalized Anxiety Disorder (GAD-7, *n* = 3) and the PHQ-4 (*n* = 1). Finally, three studies assessed the prevalence of hazardous drinking using the Alcohol Use Disorder Identification Test (AUDIT, *n* = 2) and the Cut Annoyed Guilty and Eye (CAGE, *n* = 1). All studies in this review used self-reported screening measures (e.g. questionnaires, scales) to determine psychiatric symptoms. Nearly all studies included ‘cut-off’ scores to assess the prevalence of probable PTSD (*n* = 11), depression (*n* = 7), anxiety (*n* = 4) and hazardous drinking (*n* = 3). Only one study used the PTSD cluster scoring criteria.

The individual prevalence of PTSD in CHDs varied from 3.5% to 44.0%, depression from 15.5% to 64.4%, anxiety from 7.0% to 35.0% and hazardous drinking varied from 6.3% to 40.7%. The results from the meta-analysis indicated that CHDs had a pooled estimated prevalence of PTSD of 17.8% (95% CI 12.4–24.0%, *n* = 12), depression of 28.2% (95% CI 20.7–36.2%, *n* = 7), anxiety of 17.2% (95% CI 6.6–31.5%, *n* = 4) and hazardous drinking of 17.8% (95% CI 6.9–32.2%, *n* = 3). The estimates from these studies demonstrated high heterogeneity between the four mental health outcomes (range *I*^2^ = 91.6–95.3%; range *τ*^2^ = 0.1–0.08; all with a *P ≤* 0.001) and moderate variation in estimate intervals. Please see Figure 2 (available as Supplementary data at *Occupational Medicine* online) for the pooled prevalence of PTSD, depression, anxiety and hazardous drinking.

We ran sensitivity analyses to investigate the impact of each individual study on the overall pooled prevalence of PTSD, depression, anxiety and hazardous drinking. In PTSD, the individual exclusion of studies affected the overall pooled prevalence estimate by 1.9%. The overall pooled prevalence estimate of PTSD without the Pierce [3] study increased to 19.7% (95% CI 14.7–25.2%; *I*^2^ = 89.9%; *P ≤* 0.001), and without the Birze [49] study decreased to 16.0% (95% CI 10.9–21.9%, *I*^2^ = 92.7%; *P ≤* 0.001). In depression, the individual exclusion of studies affected the overall pooled prevalence estimates by 3.9%. The overall pooled prevalence estimate of depression without the Regehr [25] study increased to 30.4% (95% CI 22.4–39.0%; *I*^2^ = 91.7%; *P* ≤ 0.001), or without the Abid [40] study decreased to 24.3% (95% CI 18.0–31.2%; *I*^2^ = 89.5%; *P* ≤ 0.001). In anxiety, the individual exclusion of studies affected the overall pooled prevalence estimates by 4.8%. The overall pooled prevalence of anxiety without the O’Dare [53] study increased to 19.2% (95% CI 6.7–36.1%; *I*^2^ = 96.4%; *P* ≤ 0.001), or without the Blalock [57] study decreased to 12.4% (95% CI 6.3–20.0%; *I*^2^ = 67.3%; *P* ≤ 0.05). In hazardous drinking, the individual exclusion of studies affected the overall pooled prevalence estimates by 7.7%. The overall pooled prevalence of hazardous drinking without the Blalock [57] study decreased to 10.1% (95% CI 7.0–13.7%, *I*^2^ and *P* were not calculated). The individual exclusion of studies did not influence the between-study heterogeneity in PTSD and depression (range *I*^2^ = 89.5–93.5%; all *P* ≤ 0.001), which reflected ‘considerable heterogeneity’ (75–100%). However, in anxiety, the exclusion of the Blalock [57] study did influence the between-study heterogeneity (*I*^2^ = 67.3%; *P* ≤ 0.05), which reflected ‘substantial heterogeneity’ (50–90%). Please see Table 5 (available as Supplementary data at *Occupational Medicine* online) for the sensitivity analysis of PTSD, depression, anxiety and hazardous drinking.

We ran separate meta-analyses of PTSD, depression, anxiety and hazardous drinking by study characteristics, sample characteristics and outcome characteristics. For example, while we noticed observable differences in the pooled prevalence estimates of PTSD by continent (North America = 19.8% [95% CI 13.4–27.1%] versus Europe = 11.2% [95% CI 8.3–14.5%]) or by type of response (emergency response = 14.2% [95% CI 8.2–21.5%] versus emergency and urgent response = 44.4% [95% CI 33.5–55.9%]), the subgroup analyses and meta-regressions were both non-significant (adjusted *R*^2^ = 28.5%, *P* = 0.3 and adjusted *R*^2^ = 3.8%, *P* = 0.1, respectively). A similar trend was also observed in other meta-analyses of depression, anxiety and hazardous drinking. Overall, all our separate subgroup analyses and meta-regression did not find evidence that any study characteristics (e.g. year of publication, continent), sample characteristics (e.g. mean age, females) and outcome characteristics (e.g. type of questionnaire), explained the high heterogeneity in the PTSD, depression, anxiety and hazardous drinking prevalence estimates. Please see Table 6 (available as Supplementary data at *Occupational Medicine* online) for the meta-analysis, subgroup analyses and meta-regressions of PTSD, depression, anxiety and hazardous drinking prevalence.

The visual inspection of the PTSD, depression and anxiety funnel plot indicated minimal evidence of asymmetry. Eggers’s regression test indicated evidence of publication bias to PTSD (*P* = 0.03) and anxiety (*P* = 0.07), but not depression (*P* = 0.6). The visual inspection of the hazardous drinking funnel plot does not indicate evidence of asymmetry. Eggers’s regression test does not indicate evidence of publication bias (*P* = 1.0). We opted not to use the trim-and-fill method for each variable, as several studies have shown that substantial heterogeneity can impact the power of the trim-and-fill method [58]. Please see Figures 3-6 (available as Supplementary data at *Occupational Medicine* online) for the funnel plot analyses of studies reporting all variables among CHDs.

## Discussion

This systematic review and meta-analysis aimed to review the available literature on mental health problems within CHDs. The extracted data from the CHD meta-analysis showed an estimated pooled prevalence (with 95% CI) of PTSD of 17.8% (95% CI 12.4–24.0%), depression of 28.2% (95% CI 20.7–36.2%), anxiety of 17.2% (95% CI 6.6–31.5%) and hazardous drinking of 17.8% (95% CI 6.9–32.2%). In general, these findings tentatively indicate a higher prevalence of PTSD and depression in CHDs when compared with other frontline first responder professionals. For example, three previous meta-analyses conducted with frontline first responders (e.g. ambulance, firefighters and police professionals) showed a pooled prevalence of PTSD ranging between 10.0% (95% CI 8.1–11.9%) and 14.2% (95% CI 10.3–18.7%) and depression between 14.6% (95% CI 10.9–18.6%) and 15% (95% CI 10.0–20.0%) [5,6,59], compared to the pooled prevalence of 17.8% (95% CI 12.4–24.0%) and 28.2% (95% CI 20.7–36.2%) found by the current study. The estimated pooled prevalence in our study is also higher than those observed in the general population, including the World Health Organization (WHO) World Mental Health Surveys, which showed a lifetime prevalence of PTSD of 3.9% and depression of 10.6% [60]. Because anxiety and hazardous drinking had wide confidence intervals in our meta-analysis, we opted not to compare values between CHDs and frontline first responders or the general population, as such comparisons could be misleading [61].

Comparing different occupational groups and/or the general population requires careful consideration, and it should be noted that some of the confidence intervals do overlap. First, the type of instruments used (e.g. self-reported measures versus ‘gold-standard’ clinical interviews) can potentially help explain the differences in prevalence estimates. For example, a meta-analysis from Levis and colleagues [62], comparing prevalence estimates in depression studies, found that self-reported studies reported higher prevalence estimates when compared with clinical interviews. Berger and colleagues [59], in their meta-analysis with frontline first responders professionals, did compare prevalence estimates in PTSD via self-reported measures versus clinical interviews, but found minimal differences. Their results showed a prevalence estimate of PTSD via self-reported measures of 10.1% (95% CI 8.1–12.2%, *n* = 35) and clinical interviews of 9.3% (95% CI 2.4–16.3%, *n* = 5); it is important to emphasize that the clinical interview group included only five studies and reported a wider confidence interval [59]. We hypothesize that the high prevalence of PTSD and depression in our meta-analysis could potentially be explained by only including self-reported measures. However, as explained above, another meta-analysis found minimal difference when comparing self-reported measures versus clinical interviews [59]. Second, studies also tend to show differences in prevalence estimates between occupational and population-based studies. For example, a meta-analysis from Goodwin and colleagues [63], found a higher prevalence of common mental disorders in occupational studies (23.9%; 95% CI 20.5–27.4%) when compared with population studies (19.2%; 95% CI 17.1–21.3%). We hypothesize that this could be the case observed here, as we are only considering CHD professionals.

Our findings tentatively indicate an elevated risk of PTSD and depression in CHDs when compared with frontline first responders or the general population. We hypothesize that the observed prevalence of mental health problems in CHDs could be explained by the specific characteristics of CHD work and their constant exposure to stress and pressure experienced at each shift. These professionals are the first point of contact in an emergency, and because they answer dozens of distressing calls every shift (e.g. suicidal callers, life-threatening illness), the cumulative stress and the constant strong negative emotions associated, might result in higher rates of PTSD and depression [3]. At the same time, CHDs do not have face-to-face contact with those they assist, and so do not receive the positive reactions that frontline first responders can sometimes benefit from, such as direct thanks and praise [64]. Additionally, CHDs initiate the emergency/urgent response, however, they do not know the final outcome, and normally, when they do, it is via a complaint or an investigation to assess responsibility (e.g. coroners court due to a serious incident). This uncertainty about their work and not knowing what happened with a specific call may prevent a sense of closure of the incident and potentially contribute to the observed high levels of anxiety, depression and PTSD [25,64].

While the observations that hazardous drinking is lower among CHDs, it is important to note first that this meta-analysis comprises only three studies, and the confidence intervals are very broad, making comparisons somewhat difficult. Studies with frontline first responders are predominantly constituted of more males, and male sex is typically associated with higher rates of alcohol abuse or hazardous drinking [5]. Thus, we hypothesize that the lower prevalence of hazardous drinking in CHD professionals might be due to the higher proportion of female professionals.

The pooled prevalence of depression was slightly higher than the pooled prevalence of PTSD and anxiety in CHDs, however, it is important to notice some overlap of confidence intervals between all variables. Depression is the most common mental disorder in society, including frontline first responders, and this review indicates the same findings with CHDs [5, 65].

The strengths of this review, includes first, the publication of a comprehensive protocol before the commencement of the review, with deviation from the original protocol on the PROSPERO database being acknowledged and reported [8]. Second, rigorous adherence to current PRISMA Guidelines that included searching ten different databases and grey literature, hand-searching the AEDR journal, identifying studies in various languages (e.g. English, Portuguese, Spanish, Italian and French), employing a well-defined criterion for study eligibility (e.g. PICOS criteria), and using validated methodological quality assessment tools (e.g. NIH Quality Assessment tools) [7]. Third, in line with the recommended advice for meta-analysis of prevalence, we employed a conservative approach and used random-effects meta-analyses, ran sensitivity analyses to identify the individual impact of each of our studies in the meta-analyses and ran subgroup analyses and meta-regressions to explain the heterogeneity [16].

The limitations of this review include, first, while we identified 37 quantitative studies, only 13 included prevalence estimates, which resulted in our meta-analyses being restricted to PTSD, depression, anxiety and hazardous drinking. Second, we observed a high heterogeneity (>90%), which our subgroup analyses or meta-regression could not explain. However, these findings align with previous work in this area that has tended to find high levels of heterogeneity in meta-analyses of prevalence [5,6]. Third, we noticed an absence of studies with small prevalence estimates, potentially suggesting a risk of selection bias in this population. Lastly, while we found evidence of publication bias in the PTSD and anxiety prevalence estimates, the trim-and-fill method is not sufficiently robust in the presence of high heterogeneity, which is why we did not perform this test in our meta-analysis [58].

The limitations of the included studies were, first, the strategy for the quality assessment showed that the majority of the included studies (78.4%) received a quality rating of ‘fair’, which suggests the presence of methodological issues in these studies and potential susceptibility to some bias. Second, we identified variability in the screening tools and cut-off scores to identify cases of PTSD, depression, anxiety and alcohol use. Thus, using different self-reported measures to assess PTSD, such as the PC-PTSD, the PCL or the IES-R, or using different cut-off scores, may reflect the observed variance in prevalence in our meta-analysis. A similar finding is also observed in depression, anxiety and alcohol use. Third, the majority of included studies were cross-sectional, so the observed outcomes might be due to prior CHD work experiences, including prior traumatic experiences (e.g. prior mental health problems, military service, among others). Relatedly, and particularly to PTSD, some studies, while assessing this condition, did not control for potentially traumatic experiences at work, which does not allow us to establish a relationship between the two variables.

This systematic review and meta-analysis is, to our knowledge, the most comprehensive investigation of quantitative data on the mental health and well-being of emergency and urgent CHDs worldwide. Our findings suggest an estimated pooled prevalence of PTSD of 17.8% (95% CI 12.4–24.0%), depression of 28.2% (95% CI 20.7–36.2%), anxiety of 17.2% (95% CI 6.6–31.5%) and hazardous drinking of 17.8% (95% CI 6.9–32.2%) among CHDs. The findings tentatively indicate an elevated risk of PTSD and depression in CHDs when compared with other frontline first responders or the general population. Despite these findings, further high-quality research is warranted. For instance, we noticed a lack of prospective cohort studies within our systematic review and meta-analysis. In observational research, prospective cohort studies are the gold standard [66]. Thus, the use of large-scale prospective cohort studies could help us better understand the cumulative effects of stress and trauma and how mental health (e.g. PTSD, depression, anxiety, alcohol abuse) prevalence rates change over time. The method of diagnosis of the papers included in our systematic review was exclusively via self-reported measures. While the study from Berger and colleagues [59] showed minimal differences in prevalence estimates between self-reported measures and clinical-administered interviews, further studies should consider both methods of diagnosis in studies with CHDs. In our systematic review, we also observed a lack of interventions for CHDs exposed to stress and trauma [67, 68]. Thus, further research into interventions that might benefit CHD mental health, such as PTSD, depression or anxiety, should be encouraged.

**Key learning points**

**What is already known about this subject:**

- Call-handlers and dispatchers (CHDs) are the first point of contact in an emergency/urgent situation, and their actions can have life-or-death consequences.
- Evidence shows these professionals experience vicarious trauma and are at increased risk of mental health problems, including post-traumatic stress disorder (PTSD), depression, anxiety and alcohol abuse, among others.

**What this study adds:**

- The results from this meta-analysis suggest an estimated pooled prevalence of post-traumatic stress disorder of 17.8%, depression of 28.2%, anxiety of 17.2% and hazardous drinking of 17.8% among call-handlers and dispatchers.
- These findings potentially indicate that call-handlers and dispatchers are at elevated risk of post-traumatic stress disorder and depression when compared with other frontline first responders.

**What impact this might have on practice or policy:**

- Further high-quality research is warranted, particularly prospective cohort studies, given that most identified studies were cross-sectional.
- The use of large-scale prospective cohort studies could help understand the cumulative effects of occupational stress and trauma on the mental health and well-being of call-handlers and dispatchers.

## Supplementary Material

kqae104_Supplementary_Material
