# Correction to: Systematic review and meta-analysis on the mental health of emergency and urgent call-handlers and dispatchers

**DOI:** 10.1093/occmed/kqaf058

**Published:** 2025-06-28

This is a correction to: C Osório, S Talwar, S A M Stevelink, H K Sihre, D Lamb, J Billings, Systematic review and meta-analysis on the mental health of emergency and urgent call-handlers and dispatchers, *Occupational Medicine*, 2024;, kqae104, https://doi.org/10.1093/occmed/kqae104

Since the original publication of this paper changes have been made to clarify tables and figures between the main document and the supplementary material. The new Table 3 has been added to the main document and removed from the supplementary material. Table and figure numbers have been adjusted for clarity.

**Table 3.** Included study and sample characteristics

Study characteristics	Number of studies (%)
**Year of publication** (studies = 34)^a^	
1980 to 1999	2 (5.9)
2000 to 2019	18 (52.9)
2020 to 2024	14 (41.2)
**Continent** (studies = 34)^a^	
Europe	9 (26.5)
Australia	1 (2.9)
Asia	1 (2.9)
North America	22 (64.8)
South America	1 (2.9)
**Sample size CHDs** (studies = 34)^a^	
<250	23 (67.6)
≥250	11 (32.4)
**Response rate** (studies = 34)^a^	
Not reported	15 (44.1)
<50%	6 (17.7)
≥50%	13 (38.2)
**Sample characteristics**	**Number of studies (%)**
**Age** (studies = 20)^b^	Median = 38.5 years (IQR = 7.5) IQR = 34.1–41.6 years
**Proportion of females** (studies = 30)	Median = 72.0% (IQR = 25.2) (Medium = 96 females) (10–690 females) Total = 4859
**Proportion of white ethnicity** (studies = 14)	Median = 78.4% (IQR = 25.9) (Medium = 150 white participants) (9–710 white ethnicity) Total = 3458 white ethnicity
**Years in service** (studies = 11)	Median = 11.9 years of experience (IQR = 8.9) IQR = 3.8–12.7 years of experience
**Work role** (studies = 34)	Total = 7759 CHDs
Call-handlers	8 (23.5)
Dispatchers	16 (47.1)
Call-handlers and dispatchers	8 (23.5)
Various roles (mixed roles)^c^	2 (5.9)
**Sector of employment** (studies = 23)	
Medical services	8 (34.8)
Police services	8 (34.8)
Search and rescue services	1 (4.3)
Various services (mixed services)^d^	6 (26.1)
**Type of response** (studies = 29)	
Emergency response (e.g. 999, 911)	26 (89.7)
Urgent response (e.g. 111, 101)	2 (6.9)
Emergency and urgent response	1 (3.4)
**Outcome characteristics** ^e^	**Number of studies (%)**
**Type of PTSD scale** (studies = 12)	
PCL questionnaire	7 (58.3)
IES questionnaire	3 (25.0)
PC-PTSD questionnaire	1 (8.3)
PDS questionnaire	1 (8.3)
**Type of depression scale** (studies = 7)	
BDI questionnaire	3 (42.9)
PHQ-9 questionnaire	3 (42.9)
PHQ-4 questionnaire	1 (14.3)
** Type of depression scale** (studies = 4)	
GAD-7 questionnaire	3 (75.0)
PHQ-4 questionnaire	1 (25.0)
** Type of alcohol scale** (studies = 3)	
AUDIT questionnaire	2 (66.7)
CAGE questionnaire	1 (33.3)

^a^Three studies were not included, as they contained the same sample.

^b^Studies that only included age as a numerical value. Studies with age as a categorical value were not included.

^c^Mixed roles—sample contained various professionals, including call-handlers and/or dispatchers but information is not provided.

^d^Mixed services—sample contained various services, including medical and police services.

^e^Outcome characteristics that were included in the meta-analysis.

Abbreviations: IQR—Interquartile Range; PCL—Post-Traumatic Stress Disorder Checklist; IES—Impact Event Scale; PC-PTSD—Primary Care Post-Traumatic Stress Disorder; PDS—Posttraumatic Stress Diagnostic Scale; BDI—Beck Depression Inventory; PHQ-9—Patient Health Questionnaire 9; PHQ-4—Patient Health Questionnaire 4; GAD-7—Generalised Anxiety Disorder 7; AUDIT—Alcohol Use Disorder Identification Test; CAGE—Cut, Annoyed, Guilty and Eye.

