# Supplementary material for: Systematic review and meta-analysis on the mental health of emergency and urgent call-handlers and dispatchers
Source: Occup Med (Lond). 2024 Nov 4;75(6):282–91. doi: 10.1093/occmed/kqae104 (PMC12449257; doi:10.1093/occmed/kqae104)
Supplement: kqae104_Supplementary_Material [file kqae104_supplementary_material.pdf]

Supplementary Material: Table 1 – Search strategy

**SOURCES**<br>Searched were conducted in different sources from inception until 7th January 2024	
**Electronic databases**	Medline (via Ovid), PubMed, APA PsycARTICLES (via Ovid), APA PsycEXTRA (via Ovid), APA PsycINFO (via Ovid), EMBASE (via Ovid), Web of Science, CINAHL Plus (via EBSCOhost) and Cochrane Library
**Grey literature**	Google Scholar
**Journals**	Annals of Emergency Dispatch and Response (AEDR)
**Other sources**	References from other literature reviews and journals
**SEARCH TERMS**<br>Search terms included sources based on the PICOS criteria and the combined use of MeSH terms and Boolean operators	
Call-handlers and dispatchers<br>Emergency call-handlers, emergency dispatchers, emergency communications, call centres, crisis calls	(call handl* OR dispatch* OR emergenc* call handl* OR emergenc* call handl* OR emergenc* dispatch* OR emergenc* call* cent* OR emergenc* dispatch* cent* OR emergenc* call* OR emergenc* communicat* OR Emergenc* med* communicat* OR Call* operator* OR emergenc* call* tak* OR cris?s call*)
Mental health and wellbeing<br>Stress, trauma, mental health, wellbeing, post-traumatic stress disorder, anxiety, depression, post-traumatic growth, resilience	**AND** (stress* OR trauma* OR mental health OR well being OR wellbeing OR ptsd OR post traumatic stress disorder OR anxiet* OR depressi* OR resilien* OR post trauma* adj3 growth) (MeSH exploded)
**LANGUAGE RESTRICTIONS**<br>Research team included at least one professional fluent in any of the mentioned languages	
**Language**	English, French, Spanish, Italian, Portuguese

Supplementary Material: Table 2 – Outcome of quality assessment

**Cohort and cross-sectional studies**	1) Objective research question?	2) Population clearly defined?	3) Participation at least 50%?	4) Subjects recruited from similar population?	5) Sample size justification?	6) Exposure of interest prior to outcome?	7) Sufficient time frame between exposure and outcome?	8) Different levels of exposure related to outcome?	9) Independent variable clearly defined, valid and reliable?	10) Exposure assessed more than once?	11) Dependent variable clearly defined, valid and reliable?	12) Assessors blinded to the exposure status?	13) Loss to follow-up?	14) Key potential confounders measured or adjusted?	**QUALITY RATING**
Jenkins, 1997	√	√	∇	√	∇	×	×	∇	√	×	√	√	∅	√	**FAIR**
Sprigg, et al., 2007	√	√	∇	√	∇	×	×	×	√	×	×	√	∅	±	**FAIR**
Farquharson, et al., 2012	√	√	×	√	∇	×	×	±	√	×	√	√	∅	√	**FAIR**
Pierce, et al., 2012	√	√	∇	√	∇	×	×	×	√	×	√	√	∅	×	**FAIR**
Anshel, et al., 2013	√	√	×	√	×	√	√	√	√	√	√	±	√	×	**FAIR**
Regehr, et al., 2013	√	√	±	√	∇	×	×	×	√	×	√	±	∅	√	**FAIR**
Allan, et al., 2014	√	√	×	√	∇	×	×	±	√	√	√	×	∅	√	**GOOD**
Lilly, et al., 2015	√	√	√	√	∇	×	×	∇	√	×	√	√	∅	±	**FAIR**
Meischke, et al., 2015	√	√	∇	√	∇	×	×	∇	√	×	√	√	∅	±	**FAIR**
Shakespeare-Finch, et al., 2015	√	√	√	√	∇	×	×	√	√	×	√	√	∅	√	**GOOD**
Trachik, et al., 2015	√	√	∇	√	∇	×	×	√	√	×	√	√	∅	√	**FAIR**
Miller, et al., 2017	√	√	×	√	∇	×	×	∇	√	×	√	√	∅	√	**FAIR**
Boland, et al., 2018	√	√	√	√	∇	×	×	√	√	×	√	√	∅	√	**GOOD**
Carleton, et al., 2018a	√	√	×	√	∇	×	×	±	√	×	√	√	∅	√	**GOOD**
Carleton, et al., 2018b	×	√	∇	√	∇	×	×	±	√	×	√	√	∅	√	**FAIR**
Steinkopf, et al., 2018	√	√	±	√	∇	×	×	√	√	×	√	√	∅	√	**GOOD**
Gilces, et al., 2019	√	√	±	√	∇	×	×	±	√	×	×	√	∅	×	**POOR**
Turner, et al., 2019	×	√	∇	√	∇	×	×	×	√	×	√	√	∅	±	**FAIR**
Birze, et al., 2020	√	√	∇	±	∇	×	×	±	√	×	√	×	∅	×	**FAIR**
Kindermann, et al., 2020	√	√	√	√	√	×	×	∇	√	×	√	√	∅	√	**FAIR**
Wahlgren, et al., 2020	√	√	√	√	∇	×	×	×	√	×	×	√	∅	√	**FAIR**

Chae, et al., 2021	√	√	∇	√	∇	×	×	∇	√	×	√	√	∅	√	**FAIR**
Galbraith, et al., 2021	√	√	√	√	∇	×	×	±	√	×	√	√	∅	±	**FAIR**
Hilaire-Schneider, et al., 2021	√	√	±	√	∇	×	×	√	√	×	√	√	∅	±	**FAIR**
Makara-Studzińska, et al., 2021	×	√	√	√	∇	×	×	∇	√	×	√	√	∅	±	**FAIR**
Wojciechowska, et al., 2021	√	√	√	√	∇	×	×	√	√	×	√	√	∅	√	**FAIR**
Birze, et al., 2022	√	√	∇	±	∇	×	×	±	√	×	√	±	∅	×	**FAIR**
Emeriau, et al., 2022	√	√	√	√	∇	×	×	√	√	×	√	√	∅	√	**GOOD**
Laguna, et al., 2022	√	√	±	√	∇	×	×	√	√	×	√	√	∅	√	**GOOD**
Schumann, et al., 2023	√	√	∇	√	∇	√	√	×	√	√	√	√	×	×	**FAIR**
O'Dare, et al., 2023	√	√	∇	√	∇	×	×	√	√	×	√	√	∅	√	**FAIR**
Blalock, et al., 2023	√	√	∇	√	∇	×	×	√	√	×	√	√	∅	×	**FAIR**
Park, et al., 2023	√	√	∇	√	∇	×	×	√	√	×	√	√	∅	√	**FAIR**

**Case-control studies**	1) Objective research question?	2) Population clearly defined?	3) Sample size justification?	4) Controls recruited from similar population?	5) Definitions, inclusion and exclusion criteria defined?	6) Cases clearly defined?	7) Cases and controls randomly selected?	8) Concurrent controls?	9) Exposure occurred prior to the development of condition?	10) Exposure clearly defined, valid, reliable?	11) Assessors blind to case or control?	12) Key potential confounders measured or adjusted?	**QUALITY RATING**
Robert, et al., 1988	×	√	×	×	√	√	∅	×	√	√	×	√	**FAIR**
Marks, et al., 2017	√	√	×	×	×	√	∅	×	√	√	×	×	**FAIR**
Ramey, et al., 2017	√	√	×	√	√	×	∅	×	√	√	∅	√	**FAIR**
Abid, et al., 2019	×	√	×	√	√	√	∅	×	√	√	×	×	**FAIR**

√ - Yes
× - No
∇ - Not reported
± - Cannot determine
∅ - Not applicable

Supplementary Material - Table 4: Summary of studies included in the systematic review.

Authors, year	Country of study	Study design	Sampling method	Sample size	Study main objective	Work role, type of response and sector of employment	Sex and age	Method of Diagnosis	Outcome variables	Main summary and conclusions
Roberg, et al., 1988	USA	Case-control	Convenience sampling	N=62 (+115 others)	Compare levels of job burnout between dispatchers and narcotics, ex-narcotics and patrol officers	Dispatchers; NP; police services	61.3% female; mean age=35.0	Self-reported measures	Burnout (MBI)	Narcotic officers reported more burnout than dispatchers. Dispatchers reported more burnout than patrol officers and ex-narcotics.
Jenkins, 1997	USA	Cross-sectional	Convenience sampling	N=68	Study the acute stress levels, coping responses, social network and distress after the Andrew Hurricane.	Dispatchers; emergency response; various services	92.6% female; mean age=37.6	Self-reported measures	PTSD (IES); Stress (IQ); Coping Styles (WCQ); Psychological Distress (BSI)	Dispatchers who worked during the Hurricane did not experience more distress than those who were at home. Dispatchers, who received CISD reported more avoidance symptoms.
Sprigg, et al., 2007	UK	Cross-sectional	Convenience sampling	N=48	Assess the prevalence and causes of verbal abuse and organisational commitment.	Call-handlers; emergency response; medical services	81.0% female; mean age=33.0	Self-reported measures	Psychological Distress (GHQ)	Verbal abuse was 7.0%. The most common sources were patients or other emergency callers. Verbal abuse was linked with poorer mental health and a desire to leave service.
Farquharson, et al., 2012 *[(a)]	UK	Cross-sectional	Convenience sampling	N=152	Explore stress levels, impact on performance, sickness absence and intentions to leave employment.	Call-handlers; urgent response; medical services	93.4% female; mean age=44.0	Self-reported measures	Psychological distress (GHQ-12); Feelings/ Emotions (PANAS); Job Satisfaction (JSS); Conflict Work/Family (WFCS)	Work-family conflict predicted intention to leave employment, sickness absence and job satisfaction.
Pierce, et al., 2012	USA	Cross-sectional	Convenience sampling	N=171	Investigate work-related trauma exposure, peritraumatic distress and PTSD.	Call-handlers; emergency response; NP	73.7% female; mean age=38.9	Self-reported measures	PTSD (PDS); Peritraumatic Distress (PDI)	PTSD was 3.5%. Peritraumatic distress was associated with PTSD severity.

Authors, year	Country of study	Study design	Sampling method	Sample size	Study main objective	Work role, type of response and sector of employment	Sex and age	Method of Diagnosis	Outcome variables	Main summary and conclusions
Anshel, et al., 2013	USA	Cohort	Convenience sampling	N=9	Determine the effectiveness of a program that offers wellness training and coping skills on changes in perceived energy, stress and job satisfaction.	Dispatchers; emergency response; police services	NP; mean age=34.8	Self-reported measures	Stress (PSS); Job Satisfaction (JS); Coping Styles (CSAS); Physical Energy (PPE)	Perceived stress, but not job satisfaction, have significantly improved in the end of program. Greater use of avoidance work-related coping strategies, but not approach coping strategies, were also observed after the end of the program. Perceived physical energy also improved after the end of the program.
Regehr, et al., 2013	Canada	Cross-sectional	Convenience sampling	N=113	Study psychological distress and physiological stress, and factors predictive of stress and distress responses.	Call-handlers; NP; police services	86.7% female; mean age=40.9	Self-reported measures	PTSD (IES-R); Depression (BDI-II); Anxiety (STAI); Coping Styles (CISS); Feelings/ Emotions (ICI); Social Support (SPS);	PTSD was 31.0%, and depression was 16.0%. PTSD and depression increased with years of employment. Emotion-focused coping predicted PTSD, depression and anxiety. Social support was negatively associated with depression.
Allan, et al., 2014 *[(a)]	UK	Cross-sectional	Convenience sampling	N=152	Study the impact of stress on cognitive failure, accuracy of information processing and speed.	Call-handlers; urgent response; medical services	92.8% female; mean age=41.0	Self-reported measures	Psychological distress (GHQ-12); Feelings/ Emotions (PANAS); Job Satisfaction (JSS)	Higher levels of stress were associated with higher memory, attention and concentration deficits.
Lilly, et al., 2015	USA	Cross-sectional	Convenience sampling	N=808	Investigate how indirect exposure affects mental health and identify factors for prevention and intervention.	Call-handlers; emergency response; NP	73.6% female; mean age=39.8	Self-reported measures	PTSD (PCL-C); Depression (BDI-II); Peritraumatic Distress (PDI & PDEQ); Personality Traits (BFI & DERS); Anger (STAXI-2); Psychological Flexibility (AAQ-II)	PTSD was 17.6% to 24.6%, and depression was 23.9%. Anger, neuroticism and event-related distress had a significant effect on psychopathology.

Authors, year	Country of study	Study design	Sampling method	Sample size	Study main objective	Work role, type of response and sector of employment	Sex and age	Method of Diagnosis	Outcome variables	Main summary and conclusions
Meischke, et al., 2015	USA	Cross-sectional	Convenience sampling	N=154	Examine stress, sources of stress, and potential stress moderators	CHDs; emergency response; NP	78.1% female; NA	Self-reported measures	Stress (C-SOSI & TCS); Job Satisfaction (ERI); Mindfulness (MAAS)	Overcommitment at work was positively associated with higher levels of stress.
Shakespeare-Finch, et al., 2015	Australia	Cross-sectional	Convenience sampling	N=60	Study the impact of self-efficacy, social support, PTG, and PTSD.	Dispatchers, emergency response; medical services	68.3% female; NA	Self-reported measures	PTSD (IES-R); PTG/Resilience (PTGI); Wellbeing (PWS); Social Support (2way SSS); Self-Efficacy (NGSE)	Receiving social support was positively predictive of wellbeing and PTG and negatively predictive of PTSD. Self-efficacy was positively predictive of wellbeing.
Trachik, et al., 2015	USA	Cross-sectional	Convenience sampling	N=205	Investigate acute stress disorders, STS and burnout.	Dispatchers, emergency response; various services	64.6% female; NA	Self-reported measures	Stress (OSSQ & SASRQ); Psychological Distress (ProQOL 5);	ASD was 17.0%. ASD was correlated with STS and burnout.
Marks, et al., 2017	USA	Case-control	Convenience sampling	N=130 (+1822 others)	Investigate PTSD symptoms between dispatchers and military veterans.	Dispatchers; emergency response; NP	64.6% female; NA	Self-reported measures	PTSD (PCL-5)	PTSD was 10.8% for dispatchers and 11.9% for military veterans. Dispatchers experienced more PTSD avoidance-related and intrusive symptoms.
Miller, et al., 2017	USA	Cross-sectional	Convenience sampling	N=186	Identify factors contributing or reducing levels of STS and burnout.	Dispatchers; emergency responses; various services	75.3% female; mean age=39.9	Self-reported measures	Psychological Distress (ProQOL 5); PTG/ Resilience (BRS); Wellbeing (SPOS & PCS)	Psychological resilience, perceived organisational support and education were associated with quality of life.
Ramey, et al., 2017	USA	Case-control	Convenience sampling	N=19 (+38 others)	Understand reported stress between CHDs and police officers.	CHDs; emergency response; police services	24.0% female; mean age=46.4	Self-reported measures	PTSD (IES); Stress (PSS); Feelings/ Emotions (POQA); PTG/ Resilience (RSES)	CHDs experienced excessive levels of chronic disease and stress. CHDs experienced higher levels of stress than police officers.

Authors, year	Country of study	Study design	Sampling method	Sample size	Study main objective	Work role, type of response and sector of employment	Sex and age	Method of Diagnosis	Outcome variables	Main summary and conclusions
Boland, et al., 2018	USA	Cross-sectional	Convenience sampling	N=19	Assess the prevalence of burnout, and the association between burnout and other factors.	Dispatchers; emergency services, medical services	NP	Self-reported measures	Burnout (MBI);	Burnout was 32.0%. No association between cumulative career exposure and burnout.
Carleton, et al., 2018a *[(b)]	Canada	Cross-sectional	Convenience sampling	N=270	Understand the prevalence of mental disorders.	CHDs, NP, various services	NP	Self-reported measures	PTSD (PCL-5); Depression (PHQ-9); Anxiety (GAD-7); Social Anxiety (SIPS); Panic Disorder (PDSS); Alcohol/drugs (AUDIT)	PTSD was 18.3%, depression was 33.2%, anxiety was 18.0%, social anxiety was 16.9%, panic disorder was 7.6% and alcohol abuse was 7.2%.
Carleton, et al., 2018b *[(b)]	Canada	Cross-sectional	Convenience sampling	N=270	Study comorbidity between mental disorders and chronic pain.	CHDs, NP, various services	NP	Self-reported measures	PTSD (PCL-5); Depression (PHQ-9); Anxiety (GAD-7); Social Anxiety (SIPS); Panic Disorder (PDSS); Alcohol/drugs (AUDIT); Chronic Pain (IASP)	Chronic pain with any mental disorders were, PTSD 50.0%, depression 46.8%, anxiety 47.7%, social anxiety 57.1%, panic disorder 41.2% and alcohol 29.4%.
Steinkopf, et al., 2018	USA	Cross-sectional	Convenience sampling	N=90	Assess operational and organisational stress, trauma, stress related-disorders and protective factors.	Dispatchers, emergency response, police services	88.9% female; NA	Self-reported measures	PTSD (PCL-C); Stress (JSS); Psychological Distress (BSI); PTG/Resilience (DRS-15 & PTGI); Feelings/Emotions (PANAS); Social Desirability (MCSDS)	Stress was 24.0%, PTSD was 13.3% and 15.6%, and subthreshold PTSD was 16.7%.

Authors, year	Country of study	Study design	Sampling method	Sample size	Study main objective	Work role, type of response and sector of employment	Sex and age	Method of Diagnosis	Outcome variables	Main summary and conclusions
Abid, et al., 2019	Pakistan	Case-control	Convenience sampling	N=45(+45 others)	Compare levels of depression and its association with demographic variables between call-handlers and controls.	Call-handlers; emergency response; search and rescue services	35.6% female; mean age=28.0	Self-reported measures	Depression (BDI-II)<br>Job Satisfaction (WSQ)	Depression was 64.4% within call-handlers. Call-handlers had higher depressive scores than controls.
Gilces, et al., 2019	Venezuela	Cross-sectional	Convenience sampling	N=34	Study psychosocial risk factors, work stress and sleep disorders.	Call-handlers; emergency response; medical services	NP; mean age=35.3	Self-reported measures	Job Satisfaction (PFAQ & JCQ); Insomnia/ Sleep Disorders (PSQI & ESS)	Low-strain work was identified in 67.6%. Poor sleep was 94.1% and daytime sleepiness was 35.3%.
Turner, et al., 2019	USA	Cross-sectional	Convenience sampling	N=833	Path analyses to study the effects of work-related factors on health-related outcomes.	CHDs; emergency response; police services	84.0% female; NP	Self-reported measures	Burnout (MBI); Stress (PSS); Depression & Anxiety (DAS); Job Satisfaction (SLS-5 & WCS 9 & WLB)	Work-life balance and burnout were strong predictors of perceived stress. Additionally, burnout predicted poorer physical health, satisfaction with life and more symptoms of anxiety and depression.
Birze, et al., 2020 *[(c)]	Canada	Cross-sectional	Convenience sampling	N=25	Assess physiological changes and mental health to acute stressful work-related events.	CHDs; emergency and urgent response; police services	88.0% female; mean age=36.5	Self-reported measures	PTSD (IES-R); Stress (MGRSS & FGRSS); Feelings/Emotions (ELS-R)	Chronic forms of emotional labour, gender role stress and PTS predicted physiological responses.
Kindermann, et al., 2020	Germany	Cross-sectional	Convenience sampling	N=71	Assess the prevalence of mental health problems and associated risk factors.	CHDs; emergency response; various services	14.1% female; mean age=33.7	Self-reported measures	PTSD (PC-PTSD); Depression & Anxiety (PHQ-4); Secondary Traumatisation (QST); Attachment (RQ-2)	Severe STS was 2.8%, PTSD was 11.3%, depression was 15.5% and anxiety was 7.0%. Absence of attachment style and higher number of children were associated with STS symptoms.

Authors, year	Country of study	Study design	Sampling method	Sample size	Study main objective	Work role, type of response and sector of employment	Sex and age	Method of Diagnosis	Outcome variables	Main summary and conclusions
Wahlgren, et al., 2020	USA	Cross-sectional	Convenience sampling	N=75	Study levels of perceived stress.	Dispatchers; emergency response; various services	60.7% female; NA	Self-reported measures	Stress (PSS)	High levels of stress were 13.3%. The main reported stressors include issues with managers, work/life balance, overtime and/or schedules, health family issues and different aspects of the job.
Chae, et al., 2021	USA & Canada	Cross-sectional	Convenience sampling	N=321	Identify the individual and organisational factors associated with stress.	CHDs; emergency response; NP	81.6% female; NA	Self-reported measures	Stress (C-SOSI & GJSQ); Mindfulness (MAAS); PTG/Resilience (CD-RISK 10); Social Support (DCSQ); Commitment (ERIS)	At an organisational level, lower organisational social support and greater job requirements were associated with higher stress levels. At an individual level, lower resilience and mindfulness, and higher overcommitment were associated with higher stress levels.
Galbraith, et al., 2021	UK	Cross-sectional	Convenience sampling	N=720	Study sources of organisational stress and whether different typologies of occupational stress were associated with physical health, mental health and substance use.	CHDs; NP; police services	43.0% female; mean age=43.0	Self-reported measures	Psychological distress (GHQ-12); Alcohol/ Drugs (B-COPE-I); Conflict-Work Family (WFCS & FIW); Stress (HSE)	Higher levels of stress associated with high demands, low control and ambiguity surrounding workplace, insufficient managerial support, mental health, substance use and worse physical health.
Makara-Studzińska, et al., 2021	Poland	Cross-sectional	Convenience sampling	N=546	Assess the moderating effects in the relationship between perceived stress, self-efficacy and burnout.	CHDs; emergency response; NP	56.4% female; mean age=34.4	Self-reported measures	Burnout (LBQ); Stress (PSS); Self-Efficacy (GSES);	Higher levels of burnout were associated with shorter work experience. Higher levels of stress were associated with greater number of shifts per month.

Authors, year	Country of study	Study design	Sampling method	Sample size	Study main objective	Work role, type of response and sector of employment	Sex and age	Method of Diagnosis	Outcome variables	Main summary and conclusions
Hilaire-Schneider, et al., 2021	France	Cross-sectional	Convenience sampling	N=283	Study the impact of day-to-day work on mental health.	Dispatchers, emergency response; medical services	72.0% female; NA	Self-reported measures	PTSD (PCL-5); Psychological Distress (ProQOL 5)	PTSD was 11.0%, burnout was 3.0%, compassion fatigue was 4.0%, and none reported STS.
Wojciechowska, et al., 2021	Poland	Cross-sectional	Convenience sampling	N=66	Assess traumatic/critical life events, PTSD and alexithymia.	Dispatchers, emergency response; NP	53.0% female; mean age=31.1	Self-reported measures	PTSD (IES-R); Stress (WPSQ); Feelings/ Emotions (TAS-20)	PTSD was 15.0%. PTSD was associated with work-related stress and alexithymia – difficulty expressing feelings. Work-related stress mediated the relationship between alexithymia and the intensity of PTS.
Birze, et al., 2022 *[(c)]	Canada	Cross-sectional	Convenience sampling	N=72	Examine the relationship among perceived organisational support, emotional labour, stress and PTS.	CHDs; emergency and urgent response; police services	90.3% female; mean age=25.0	Self-reported measures	PTSD (IES-R); Stress (JSS); Feelings/ Emotions (ELS-R)	PTSD was 44.0%. PTS symptoms was associated with perceived lack of organisational support and job pressure.
Emeriau, et al., 2022	Canada	Cross-sectional	Convenience sampling	N=155	Study the influence of recognition at work on psychological health.	Dispatchers; emergency response; NP	60.4% female; NA	Self-reported measures	Psychological Distress (PHQ); Job Satisfaction (RaW)	Organisational and co-worker recognition partially explained psychological distress and psychological wellbeing at work.
Laguna, et al., 2022	Poland	Cross-sectional	Convenience sampling	N=335	Examined several theoretical aspects of job characteristics on staff wellbeing.	Dispatchers; emergency response; medical services	49.1% female; mean age=45.3	Self-reported measures	Job Satisfaction (WDQ & QWI); Wellbeing (JAWS)	The higher workload within dispatchers, the lower job-related effective wellbeing. The higher job autonomy and performance feedback, the higher affective wellbeing.

Authors, year	Country of study	Study design	Sampling method	Sample size	Study main objective	Work role, type of response and sector of employment	Sex and age	Method of Diagnosis	Outcome variables	Main summary and conclusions
Schumann, et al., 2023 **	Germany	Cohort	Convenience sampling	N=440 (t1)<br>N=50 (t2)	Study the recovery stress-status of emergency dispatchers during the first and second waves of SARS-CoV-2 pandemic.	Dispatchers; Emergency response; medical services	7.7% female (t1); mean age=42.4 (t1); 6.0% female (t2) age=40.8 (t2)	Self-reported measures	Stress (RSQ)	Stress experience increased from the first to second wave of SARS-CoV-2 pandemic, while the recovery of dispatchers decreased.
O'Dare, et al., 2023	USA	Cross-sectional	Convenience sampling	N=54	Examined the relationship between job-related traumatic events and mental health	Dispatchers; NP; NP	66.7% female; mean age=38.0	Self-reported measures	PTSD (PCL-5); Depression (PHQ-9); Anxiety (GAD-7); Alcohol (AUDIT); Suicide (SBQR)	PTSD was 14.0% or 16.0%, moderate-severe depression was 18.4%, moderate-severe anxiety was 12.0%, hazardous alcohol use was 40.0% and increased suicide risk was 9.3%.
Blalock, et al., 2023	USA	Cross-sectional	Convenience sampling	N=742	Examined the prevalence and severity of anxiety, depression, PTSD, alcohol use and suicidal ideation.	Dispatchers; Emergency response; police services	74.0% female; NA	Self-reported measures	PTSD (PCL-5); Depression (PHQ-9); Anxiety (GAD-7); Alcohol (CAGE); Suicide (C-SSRS)	PTSD was 29.3%, depression was 35.6%, anxiety was 35.0%, alcohol was 15.5% and experiencing passive thoughts of suicide was 12.4%.
Park, et al., 2023	USA & Canada	Cross-sectional	Convenience sampling	N=403	Examined the relationship between overtime hours (mandatory and voluntary) and levels of stress symptoms	Call-handlers; Emergency response; NP	81.9% female; NA	Self-reported measures	Stress (C-SOSI)	Mandatory overtime hours were associated with stress symptoms.

Abbreviations: NP – Not Provided; NA – Not Applicable (e.g. age reported as a categorical variable); PTSD – Post-Traumatic Stress Disorder; PTS – Post-Traumatic Stress; ASD – Acute Stress Disorder; STS – Secondary Traumatic Stress; PTG – Posttraumatic Growth; CISD - Critical Incident Stress Debriefing; CHDs – Call-Handlers and Dispatchers;

PTSD: IES – Impact Event Scale; IES-R – Impact Event Scale-Revised; PCL-C – Post Traumatic Stress Disorder Checklist – Civilian Version; PCL-5 – Post-Traumatic Stress Disorder Checklist 5; PC-PTSD – Primary Care PTSD; PDS - Posttraumatic Stress Diagnostic Scale; **Depression:** BDI-II – Beck Depression Inventory-II; PHQ-9 – Patient Health Questionnaire 9; PHQ-4 – Patient Health Questionnaire 4; DAS – Depression and Anxiety Scale; **Job Satisfaction:** JS – Job Satisfaction 6 item; WSQ – Workplace Stress Questionnaire; PFAQ – Psychosocial Factor Assessment Questionnaire; JCQ – Job Content Questionnaire; ERI – Effort Reward Imbalance; WLB – Work-Life Balance; WCS-9 – Work Conditions Scale 9 Item; SLS-5 – Satisfaction with Life Scale 5 Item; RaW – Recognition at Work; WDQ – Work Design Questionnaire; QWI – Quantitative Workload Inventory; **Psychological Distress:** GHS-12 - General Health Questionnaire-12; BSI – Derogatis Brief Symptom Inventory; ProQOL - Professional Quality of Life Scale; PHQ – Psychological Health at Work; **Feelings/Emotions:** PANAS - Positive and Negative Affect Schedule; ELS-R - Emotional Labour Scale-Revised; POQA - Personal and Organisational Quality Assessment; ICI – Internal Control Index; TAS-20 - Toronto Alexithymia Scale 20 items; **Stress**: MGRSS & FGRSS – Masculine/ Feminine Gender Role Stress Scale; JSS – Job Stress Inventory; C-SOSI – Calgary Symptoms of Stress Inventory; GJSQ – NIOSH Generic Job Stress Questionnaire; HSE - Health and Safety Executive; IC – Incident Questionnaire; PSS – Perceived Stress Scale; TCS - Technostress Creator Scale; OSSQ – Organisational Sources of Stress Questionnaire; SASRQ - Stanford Acute Stress Reactions Questionnaire; WPSQ - Workplace Perceived Stress Questionnaire; RSQ – Recovery Stress Questionnaire; **Coping Styles**: WCQ – Ways of Coping Questionnaire; RSQ - Recovery Stress Questionnaire; CSAS – Coping Styles for Acute Stress 24 items; **Burnout**: MBI – Maslach Burnout Inventory; LBQ – Link Burnout Questionnaire; **Anxiety**: GAD7 – Generalised Anxiety Disorder 7; PHQ-4 – Patient Health Questionnaire 4; DAS – Depression and Anxiety Scale; **Social Anxiety**: SIPS – Social Interactions Phobia Scale 14; **Panic Disorder**: PDSS – Panic Disorder Symptom Severity Scale 7; **Secondary Traumatisation**: QST – Questionnaire for Secondary Traumatisation; **Posttraumatic Growth/ Resilience**: BRS - Brief Resilience Survey; RSES – Response to Stress Event Scale; DRS-15 - Dispositional Resilience Scale 15; PTGI – Posttraumatic Growth Inventory; **Personality Traits**: BFI – Big Five Inventory; DERS – Difficulties in Emotional Regulation Scale; **Peritraumatic Distress**: PDI – Peritraumatic Distress Inventory; PDEQ – Peritraumatic Dissociative Experiences Questionnaire; **Alcohol/Drugs**: AUDIT – Alcohol Use Disorders Identification Test; CAGE- Cut, Annoyed, Guilty and Eye; **Suicide**: SBQR -Suicidal Behaviours Questionnaire Revised; C-SSRS – Columbia Suicide Severity Rating Scale: **Mindfulness**: MAAS – Mindful Attention Awareness Scale; **Wellbeing**: PCS – Perceived Co-worker Support; SPOS – Survey of Perceived Organisational Support; PWS – Psychological Wellbeing Scale 42 Item; JAWS – Job-related Affective Wellbeing Scale; **Psychological Flexibility**: AAQ-II - Acceptance and Action Questionnaire-II; **Self-Efficacy**: GSES – Generalised Self-Efficacy Scale; NGSE - New General Self-Efficacy Scale; **Social Support**: DCSQ – Demand Control Support Questionnaire; SPS – Social Provisions Scale; 2way SSS – 2 Way Social Support Scale 20 Item; **Commitment**: ERIS – Effort-Reward Imbalance Scale; **Attachment**: RQ-2 – Relationship Questionnaire 2; **Social Desirability**: **MCSDS** - Marlowe-Crowne Social Desirability Scale; **Conflict Work/Family**: WFCS – Work-Family Conflict Scale (WFCS); FIW – Family Interference with Work; **Insomnia/ Sleep Disorders**: PSQI – Pittsburgh Sleep Quality Index; ESS – Epworth Sleepiness Scale; **Chronic Pain**: IASP – Self-reported items from the International Association for the Study of Pain; **Physical Energy**: PPE - Perceived Physical Energy 13 item.

*[a, b & c] Different study with the same sample.

** Another study, Schumann et al., 2023, compared emergency dispatchers with emergency medical service personnel, but because it used the same sample of emergency dispatchers, it was not included here.

Supplementary Material - Table 5: Sensitivity Analysis of PTSD, Depression, Anxiety and Hazardous Drinking Prevalence Estimates

PTSD					
**Meta-analysis <u>WITHOUT</u> [a]**	**Pooled Prevalence**	**95%CI**	**I^2**	**P value**	**Difference to Original Meta-Analysis Value (17.8%)**
Pierce, 2012	19.7	14.7 – 25.2	89.9	≤0.001	+ 1.9
Regehr, 2013	16.8	11.3 – 23.1	93.3	≤0.001	- 1.0
Wojciechowska, 2021	18.1	12.4 – 24.6	93.5	≤0.001	+ 0.3
Birze, 2022	16.0	10.9 – 21.9	92.7	≤0.001	- 1.8
Lilly, 2015	17.2	10.9 – 24.5	93.2	≤0.001	- 0.6
Steinkopf, 2018	18.3	12.5 – 24.8	93.4	≤0.001	+ 0.5
Marks, 2017	18.6	12.8 – 25.0	93.1	≤0.001	+ 0.8
Carleton, 2018	17.7	11.8 – 24.6	93.6	≤0.001	- 0.1
Hilaire-Schneider, 2021	18.6	13.0 – 25.0	92.3	≤0.001	+ 0.8
O'Dare, 2023	18.1	12.4 – 24.6	93.5	≤0.001	+ 0.3
Blalock, 2023	16.8	11.2 – 23.2	91.6	≤0.001	- 1.0
Kindermann, 2020	18.4	12.7 – 25.0	93.4	≤0.001	+ 0.6
**Depression**					
**Meta-Analysis <u>WITHOUT</u> [a]**	**Pooled Prevalence**	**95%CI**	**I^2**	**P value**	**Difference to Original Meta-Analysis Value (28.2%)**
Regehr, 2013	30.4	22.4 – 39.0	91.7	≤0.001	+ 2.2
Lilly, 2015	29.1	19.7 – 39.5	90.9	≤0.001	+ 0.9
Abid, 2019	24.3	18.0 – 31.2	89.5	≤0.001	- 3.9
Carleton, 2018	27.4	18.7 – 37.1	92.8	≤0.001	- 0.8
O'Dare, 2023	29.6	21.5 – 38.3	92.7	≤0.001	+1.4
Blalock, 2023	26.8	18.3 – 36.3	89.6	≤0.001	- 1.4
Kindermann, 2020	30.2	22.2 – 38.8	92.2	≤0.001	+ 2.0
**Anxiety**					
**Meta-Analysis <u>WITHOUT</u> [a]**	**Pooled Prevalence**	**95%CI**	**I^2**	**P value**	**Difference to Original Meta-Analysis Value (17.2%)**
Carleton, 2018	16.7	2.4 – 39.7	95.6	≤0.001	- 0.5
O'Dare, 2023	19.2	6.7 – 36.1	96.4	≤0.001	+ 2.0
Blalock, 2023	12.4	6.3 – 20.0	67.3	≤0.05	- 4.8
Kindermann, 2020	21.2	9.1 – 36.6	95.2	≤0.001	- 4.0
**Alcohol Use**					
**Meta-Analysis <u>WITHOUT</u> [a]**	**Pooled Prevalence**	**95%CI**	**I^2**	**P value**	**Difference to Original Meta-Analysis Value (17.8%)**
Carleton, 2018	16.7	14.1 – 19.4	NC	NC	- 1.1
O'Dare, 2023	12.7	10.7 – 14.8	NC	NC	- 5.1
Blalock, 2023	10.1	7.0 – 13.7	NC	NC	- 7.7

Abbreviations: NC – Not calculated
[a] Sensitivity analysis investigated the impact of each study on the overall pooled prevalence

Supplementary Material - Figure 2. Pooled prevalence of PTSD, depression, anxiety and hazardous drinking.

Authors, year	PTSD measure	Cut-off score ≥	CHDs sample	PTSD cases	Prevalence (95%CI)	Prevalence (95%CI)	Weight, %
Pierce, 2012	PDS	28	171	6	3.5 (1.6 – 7.4)	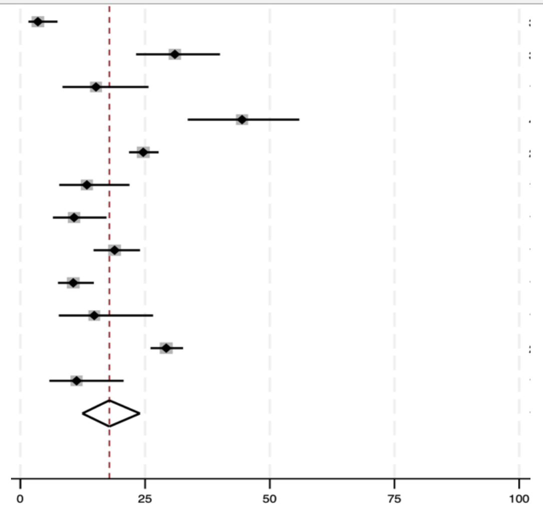	8.6
Regehr, 2013	IES-R	33	113	35	31.0 (23.2 -40.0)		8.3
Wojciechowska, 2021	IES-R	33	66	10	15.2 (8.4 – 25.7)		7.6
Birze, 2022	IES-R	33	72	32	44.4 (33.5 – 55.9)		7.7
Lilly, 2015	PCL-C	44	808	199	24.6 (21.8 – 27.7)		9.3
Steinkopf, 2018	PCL-C	44	90	12	13.3 (7.8 – 21.9)		8.0
Marks, 2017	PCL-5 *	NA	130	14	10.8 (6.5 – 17.3)		8.4
Carleton, 2018	PCL-5	32	270	51	18.9 (14.7 – 24.0)		8.9
Hilaire-Schneider, 2021	PCL-5	34	283	30	10.6 (7.5 – 14.7)		8.9
O'Dare, 2023	PCL-5	30	54	8	14.8 (7.7 – 26.6)		7.3
Blalock, 2023	PCL-5	30	742	217	29.3 (26.1 – 32.6)		9.3
Kindermann, 2020	PC-PTSD	3	71	8	11.3 (5.8 – 20.7)		7.7
**Pooled summary estimate**<br>Tests for heterogeneity: χ^2=155.8; d.f.=11 (p≤0.001); I^2=92.9%; τ^2=0.06<br>Test for overall effect: z=10.3 (p≤0.001)					**17.8 (12.4 – 24.0)**		**100**

Authors, year	Depression measure	Cut-off score ≥	CHDs sample	Depression cases	Prevalence (95%CI)	Prevalence (95%CI)	Weight, %
Regehr, 2013	BDI	NR	113	18	15.9 (10.3 – 23.8)	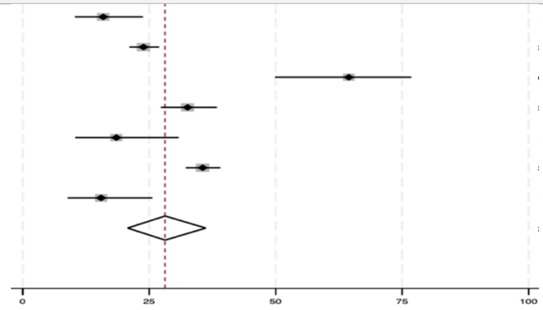	14.3
Lilly, 2015	BDI-II	NR	808	193	23.9 (21.1 – 27.0)		16.7
Abid, 2019	BDI-II	20	45	29	64.4 (49.8 – 76.8)		11.4
Carleton, 2018	PHQ-9	10	270	88	32.6 (27.3 – 38.4)		15.8
O'Dare, 2023	PHQ-9	15	54	10	18.5 (10.4 – 30.8)		12.1
Blalock, 2023	PHQ-9	10	742	264	35.6 (32.2 – 39.1)		16.6
Kindermann, 2020	PHQ-4	3	71	11	15.5 (8.9 – 25.7)		13.0
**Pooled Summary Estimate**<br>Tests for heterogeneity: χ^2=71.7; d.f.=6 (p≤0.001); I^2=91.6%; τ^2=0.04<br>Test for overall effect: z=11.8 (p≤0.001)					**28.2 (20.7 – 36.2)**		**100**

Authors, year	Anxiety measure	Cut-off score ≥	CHDs sample	Anxiety cases	Prevalence (95%CI)	Prevalence (95%CI)	Weight, %
Carleton, 2018	GAD-7	10	270	48	17.8 (13.7 – 22.8)	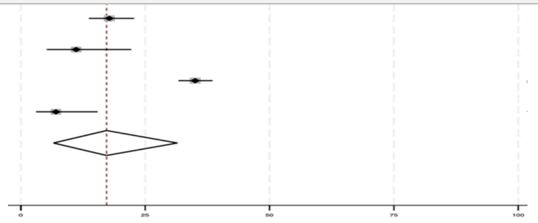	26.2
O'Dare, 2023	GAD-7	8	54	6	11.1 (5.2 – 22.2)		23.1
Blalock, 2023	GAD-7	10	742	260	35.0 (31.7 – 38.6)		26.8
Kindermann, 2020	PHQ-4	3	71	5	7.0 (3.1 – 15.5)		23.9
**Pooled Summary Estimate**<br>Tests for heterogeneity: χ^2=64.2; d.f.=3 (p≤0.001); I^2=95.3%; τ^2=0.1<br>Test for overall effect: z=4.55 (p≤0.001)					**17.2 (6.6 – 31.5)**		**100**

Authors, year	Alcohol measure	Cut-off score ≥	CHDs sample	Alcohol cases	Prevalence (95%CI)	Prevalence (95%CI)	Weight, %
Carleton, 2018	AUDIT	16	270	17	6.3 (4.0 – 9.9)	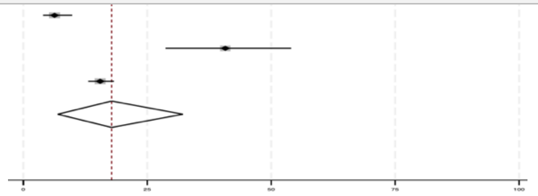	34.7
O'Dare, 2023	AUDIT	8	54	22	40.7 (26.7 – 54.0)		29.5
Blalock, 2023	CAGE	2	742	115	16.5 (13.1 – 18.3)		35.8
**Pooled Summary Estimate**<br>Tests for heterogeneity: χ^2=39.8; d.f.=2 (p≤0.001); I^2=95.0%; τ^2=0.08<br>Test for overall effect: z=4.7 (p≤0.001)					**17.8 (6.9 – 32.2)**		**100**

* Full criteria for PTSD cluster scoring (symptoms rated moderate or higher – 1 Cluster B, 1 Cluster C, 2 Cluster D, 2 Cluster E)

Abbreviations: NA – Not Applicable; NR - Not reported; PCL – Post-Traumatic Stress Disorder Checklist; IES - Impact Event Scale; PC-PTSD – Primary Care Post-Traumatic Stress Disorder; PDS – Posttraumatic Stress Diagnostic Scale; BDI – Beck Depression Inventory; PHQ-9 – Patient Health Questionnaire 9; PHQ-4 – Patient Health Questionnaire 4; GAD-7 - Generalised Anxiety Disorder 7; AUDIT – Alcohol Use Disorder Identification Test; CAGE – Cut, Annoyed, Guilty and Eye;

Supplementary Material - Table 6: Meta-analysis, subgroup analyses and meta-regressions of PTSD, depression, anxiety and hazardous drinking prevalence by study characteristics, sample characteristics and outcome characteristics.

Meta-Analysis, Subgroup Analysis and Meta-regression of PTSD by Study Characteristics, Sample Characteristics and Outcome Characteristics								
	Meta-Analysis						Subgroup Analyses and Meta-Regression	
	Number of studies	Total number of participants	Total cases	Prevalence of PTSD, % (95%CI)	χ^2	I^2 (%)	*Adjusted* R^2 (%)	*P* Value for subgroup differences
**Study characteristics**								
**Year of publication**								
≤ 2019	6	1582	317	16.0 (8.8 – 24.7)	77.3	93.5	-39.4	0.6
≥ 2020	6	1288	305	19.9 (10.9 – 30.8)	72.7	93.1		
**Continent**								
North America	9	2450	574	19.8 (13.4 – 27.1)	122.6	93.5	28.5	0.3
Europe	3	420	48	11.2 (8.3 – 14.5)	NC	NC		
**Sample size CHDs**								
< 250	8	767	125	16.5 (8.4 – 26.5)	79.9	91.2	-8.4	0.6
≥ 250	4	2103	497	20.6 (13.7 – 28.4)	50.3	94.0		
**Response rate**								
Not reported	5	1210	280	16.4 (6.1 – 30.4)	97.5	95.9	-46.7	0.7
< 50%	2	360	63	17.4 (13.6 – 21.5)	NC	NC		
≥ 50%	5	1300	279	20.0 (11.1 – 30.6)	53.6	92.5		
**Sample characteristics**								
**Age** [a]	7	1355	298	19.1 (10.2 – 30.0)	91.9	93.5	-44.4	0.7
**Female** [b]	10	2529	563	18.4 (12.1 – 25.7)	150.4	94.0	2.1	0.6
**White ethnicity** [c]	4	1775	430	16.9 (7.9 – 28.5)	81.8	96.3	5.1	0.8
**Years in service**	5	1229	258	15.9 (6.6 – 28.0)	71.5	94.4	0.4	0.7
**Work role**								
Call-handlers	3	1092	240	17.6 (4.5 – 36.7)	NC	NC	-25.4	0.7
Dispatchers	6	1365	291	15.4 (8.1 – 24.4)	65.6	92.4		
Call-handlers and dispatchers	2	143	40	26.3 (19.3 – 33.9)	NC	NC		
Various roles (mixed roles) [d]	1	270	51	18.9 (14.7 – 24.0)	NC	NC		
**Sector of employment**								
Medical Services	1	283	30	10.6 (7.5 – 14.7)	NC	NC	-27.4	0.8
Police services	4	1017	296	28.7 (19.4 – 38.8)	20.8	85.6		
Various services (mixed services) [e]	2	341	59	17.1 (13.2 – 21.3)	NC	NC		

**Type of response**								
Emergency response (e.g. 999, 911)	8	2361	496	14.2 (8.2 – 21.5)	128.3	94.5	3.8	0.1
Emergency and urgent response	1	72	32	44.4 (33.5 – 55.9)	NC	NC		
**Outcome Characteristics**								
**Type of PTSD questionnaire**								
PCL questionnaire	7	2377	531	17.5 (12.1 – 23.6)	69.8	91.4	-3.8	0.3
IES questionnaire	3	251	77	29.6 (15.3 – 46.1)	NC	NC		
PDS questionnaire	1	171	6	3.5 (1.6 – 7.4)	NC	NC		
PC-PTSD questionnaire	1	71	8	11.3 (5.8 – 20.7)	NC	NC		

**Meta-Analysis, Subgroup Analysis and Meta-regression of Depression by Study Characteristics, Sample Characteristics and Outcome Characteristics**								
	**Meta-Analysis**						**Subgroup Analyses and Meta-Regression**	
	**Number of studies**	**Total number of participants**	**Total cases**	**Prevalence of Depression, % (95%CI)**	**χ^2**	**I^2 (%)**	**Adjusted R^2 (%)**	***P* Value for subgroup differences**
**Study characteristics**								
**Year of publication**								
≤ 2019	4	1236	328	31.7 (20.3 – 44.4)	42.4	92.9	-60.8	0.6
≥ 2020	3	867	285	23.4 (10.8 – 39.1)	NC	NC		
**Continent**								
North America	5	1987	573	25.8 (19.1 – 33.2)	40.9	90.2	3.8	0.3
Europe	1	71	11	15.5 (8.9 – 25.7)	NC	NC		
South Asia	1	45	29	64.4 (49.8 – 76.8)	NC	NC		
**Sample size CHDs**								
< 250	4	283	68	26.7 (10.0 – 47.8)	39.5	92.4	-10.6	0.6
≥ 250	3	1820	545	30.5 (22.7 – 38.9)	NC	NC		
**Response rate**								
Not reported	3	909	292	23.4 (10.7 – 39.1)	NC	NC	-60.7	0.6
< 50%	1	270	88	32.6 (27.3 – 38.4)	NC	NC		
≥ 50%	3	924	233	32.5 (13.4 – 55.3)	NC	NC		
**Sample characteristics**								
**Age** [a]	5	1091	261	25.7 (15.2 – 37.9)	39.6	89.9	0.6	0.4
**Female** [b]	4	1717	485	23.9 (15.9 – 33.0)	38.6	92.2	-6.4	0.5
**White ethnicity** [c]	3	1604	467	26.7 (17.7 – 36.8)	NC	NC	NC	NC
**Years in service**	3	992	222	19.4 (13.6 – 25.8)	NC	NC	-30.4	0.6

**Work role**								
Call-handlers	3	966	240	32.3 (14.8 – 52.7)	NC	NC	-28.5	0.9
Dispatchers	2	796	274	34.2 (30.9 – 37.6)	NC	NC		
Call-handlers and dispatchers	1	71	11	15.5 (8.9 – 25.7)	NC	NC		
Various roles (mixed roles) [d]	1	270	88	32.6 (27.3 – 38.4)	NC	NC		
**Sector of employment**								
Police Services	2	855	282	32.7 (29.6 – 35.9)	NC	NC	-108.4	0.4
Search and Rescue services	1	45	29	64.4 (49.8 – 76.8)	NC	NC		
Various services (mixed services) [e]	2	341	99	28.7 (24.0 – 33.6)	NC	NC		
**Type of response**								
Emergency response (e.g. 999, 911)	3	1621	468	25.5 (16.3 – 35.9)	NC	NC	29.3	0.2
Urgent response (e.g. 111, 101)	1	45	29	64.4 (49.8 – 76.8)	NC	NC		
**Outcome Characteristics**								
**Type of Depression questionnaire**								
BDI questionnaire	3	966	240	32.3 (14.8 – 52.7)	NC	NC	-42.2	0.6
PHQ-9 questionnaire	3	1066	362	31.1 (24.5 – 38.1)	NC	NC		
PHQ-4 questionnaire	1	71	11	15.5 (8.9 – 25.7)	NC	NC		

Meta-Analysis, Subgroup Analysis and Meta-regression of Anxiety by Study Characteristics, Sample Characteristics and Outcome Characteristics

	Meta-Analysis						Subgroup Analyses and Meta-Regression	
	**Number of studies**	**Total number of participants**	**Total cases**	**Prevalence of Anxiety, % (95%CI)**	χ^2	***I*² (%)**	***Adjusted R*² (%)**	***P* Value for subgroup differences**
**Year of publication**								
≤ 2019	1	270	48	17.8 (13.7 – 22.8)	NC	NC	-152.0	0.9
≥ 2020	3	867	271	16.7 (2.4 – 39.7)	NC	NC		
**Continent**								
North America	3	1066	314	21.2 (9.1 – 36.6)	NC	NC	24.7	0.3
Europe	1	71	5	7.0 (3.1 – 15.5)	NC	NC		
**Sample size CHDs**								
< 250	2	125	11	8.7 (4.2 – 14.4)	NC	NC	41.6	0.2
≥ 250	2	1012	308	30.1 (27.3 – 33.0)	NC	NC		
**Response rate**								
Not reported	2	796	266	33.1 (29.8 – 36.4)	NC	NC	46.7	0.3
< 50%	1	270	48	17.8 (13.7 – 22.8)	NC	NC		
≥ 50%	1	71	5	7.0 (3.1 – 15.5)	NC	NC		

**Sample characteristics**								
**Age** [a]	2	125	11	8.7 (4.2 – 14.4)	NC	NC	41.6	0.2
**Female** [b]	2	796	266	33.1 (29.8 – 36.4)	NC	NC	32.6	0.5
**White ethnicity** [c]	2	796	266	33.1 (29.8 – 36.4)	NC	NC	NC	NC
**Years in service**	1	71	5	7.0 (3.1 – 15.5)	NC	NC	NC	NC
**Work role**								
Dispatchers	2	796	266	33.1 (29.8 – 36.4)	NC	NC	-109.6	0.8
Call-handlers and dispatchers	1	71	5	7.0 (3.1 – 15.5)	NC	NC		
Various roles (mixed roles) [d]	1	270	48	17.8 (13.7 – 22.8)	NC	NC		
**Sector of employment**								
Police Services	1	742	260	35.0 (31.7 – 38.5)	NC	NC	1.5	0.4
Various services (mixed services) [e]	2	341	53	15.2 (11.5 – 19.2)	NC	NC		
**Type of response**								
Emergency response (e.g. 999, 911)	2	813	265	32.0 (28.8 – 35.3)	NC	NC	NC	NC
**Outcome Characteristics**								
**Type of Anxiety questionnaire**								
GAD-7 questionnaire	3	1066	314	21.2 (9.1 – 36.6)	NC	NC	24.7	0.3
PHQ-4 questionnaire	1	71	5	7.0 (3.1 – 15.5)	NC	NC		

**Meta-Analysis, Subgroup Analysis and Meta-regression of Hazardous Drinking by Study Characteristics, Sample Characteristics and Outcome Characteristics**								
	**Meta-Analysis**						**Subgroup Analyses and Meta-Regression**	
	**Number of studies**	**Total number of participants**	**Total cases**	**Prevalence of Hazardous Drinking, % (95%CI)**	**χ^2**	**I^2 (%)**	***Adjusted R^2* (%)**	***P* Value for subgroup differences**
**Year of publication**								
≤ 2019	1	270	17	6.3 (4.0 – 9.9)	NC	NC	12.9	0.4
≥ 2020	2	796	137	16.7 (14.1 – 19.4)	NC	NC		
**Continent**								
North America	3	1066	154	17.8 (6.9 – 32.2)	NC	NC	NC	NC
**Sample size CHDs**								
< 250	1	54	22	40.7 (28.7 – 54.0)	NC	NC	25.2	0.3
≥ 250	2	1012	132	12.7 (10.7 – 14.8)	NC	NC		
**Response rate**								
Not reported	2	796	137	16.7 (14.1 – 19.4)	NC	NC	12.9	0.4
< 50%	1	270	17	6.3 (4.0 – 9.9)	NC	NC		

**Sample characteristics**								
**Age** [a]	1	54	22	40.7 (28.7 – 54.0)	NC	NC	25.2	0.3
**Female** [b]	2	796	137	16.7 (14.1 – 19.4)	NC	NC	NC	NC
**White ethnicity** [c]	2	796	137	16.7 (14.1 – 19.4)	NC	NC	NC	NC
**Years in service**	ND	ND	ND	ND	ND	ND	NC	NC
**Work role**								
Dispatchers	2	796	137	16.7 (14.1 – 19.4)	NC	NC	12.9	0.4
Various roles (mixed roles) [d]	1	270	17	6.3 (4.0 – 9.9)	NC	NC		
**Sector of employment**								
Police Services	1	742	115	15.5 (13.1 – 18.3)	NC	NC	NC	NC
Various services (mixed services) [e]	1	270	17	6.3 (4.0 – 9.9)	NC	NC		
**Type of response**								
Emergency response (e.g. 999, 911)	1	742	115	15.5 (13.1 – 18.3)	NC	NC	NC	NC
**Outcome Characteristics**								
**Type of Alcohol Use questionnaire**								
AUDIT questionnaire	2	324	39	10.1 (7.0 – 13.7)	NC	NC	-240.4	1.0
CAGE questionnaire	1	742	115	15.5 (13.1 – 18.3)	NC	NC		

[a] Age – mean years
[b] Proportion of female
[c] Proportion of white ethnicity
[d] Mixed roles – sample contained various professionals, including call-handlers and/or dispatchers but information is not provided.
[e] Mixed services – sample contained various services, including medical and police services.

Abbreviations: NC – Not calculated; ND – No data available; PTSD – Post-Traumatic Stress Disorder; CHDs – Call-Handlers and Dispatchers; PCL – Post-Traumatic Stress Disorder Checklist; IES - Impact Event Scale; PC-PTSD – Primary Care Post-Traumatic Stress Disorder; PDS – Posttraumatic Stress Diagnostic Scale; BDI – Beck Depression Inventory; PHQ-9 – Patient Health Questionnaire 9; PHQ-4 – Patient Health Questionnaire 4; GAD-7 - Generalised Anxiety Disorder 7; AUDIT – Alcohol Use Disorder Identification Test; CAGE – Cut, Annoyed, Guilty and Eye;

Supplementary Material: Figure 3 - Funnel plot assessment of studies reporting PTSD among CHDs

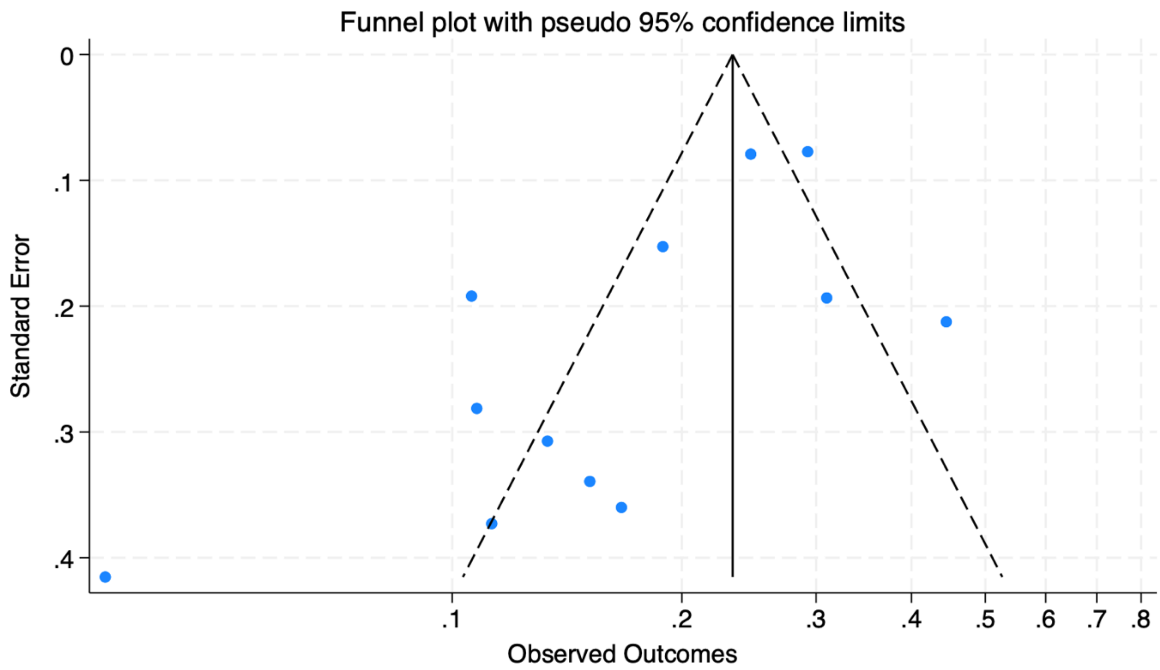

Egger's regression test (p=0.03)

Supplementary Material: Figure 4 - Funnel plot assessment of studies reporting Depression among CHDs

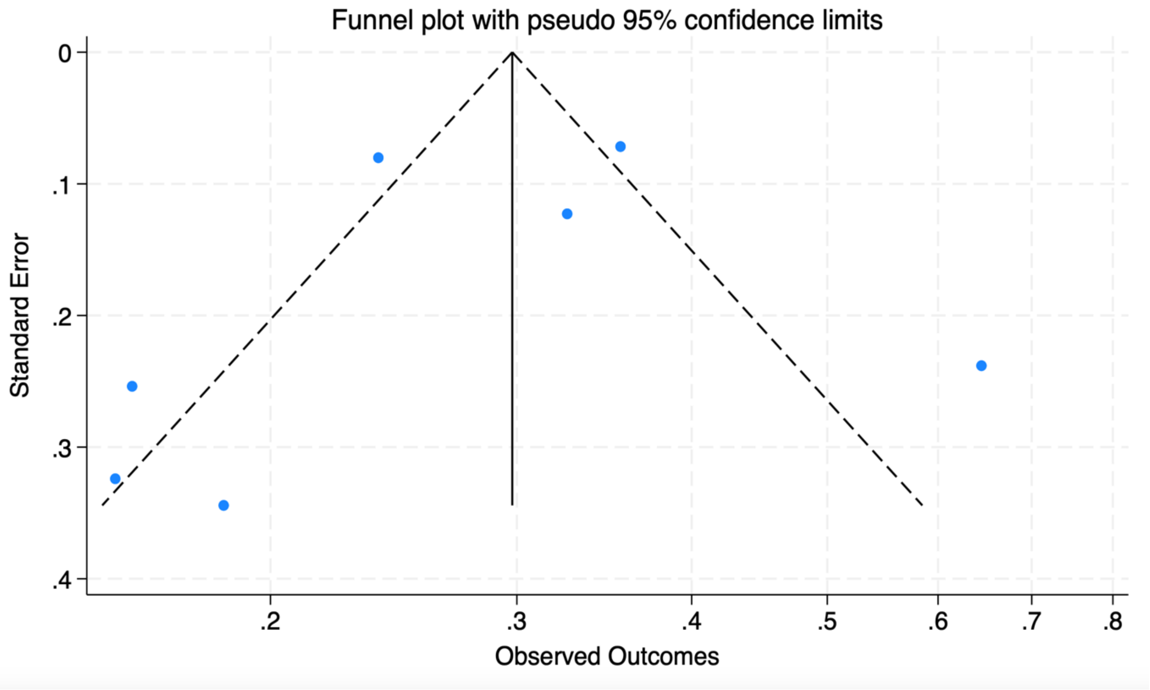

Egger's regression test (p=0.6)

Supplementary Material: Figure 5 - Funnel plot assessment of studies reporting Anxiety among CHDs

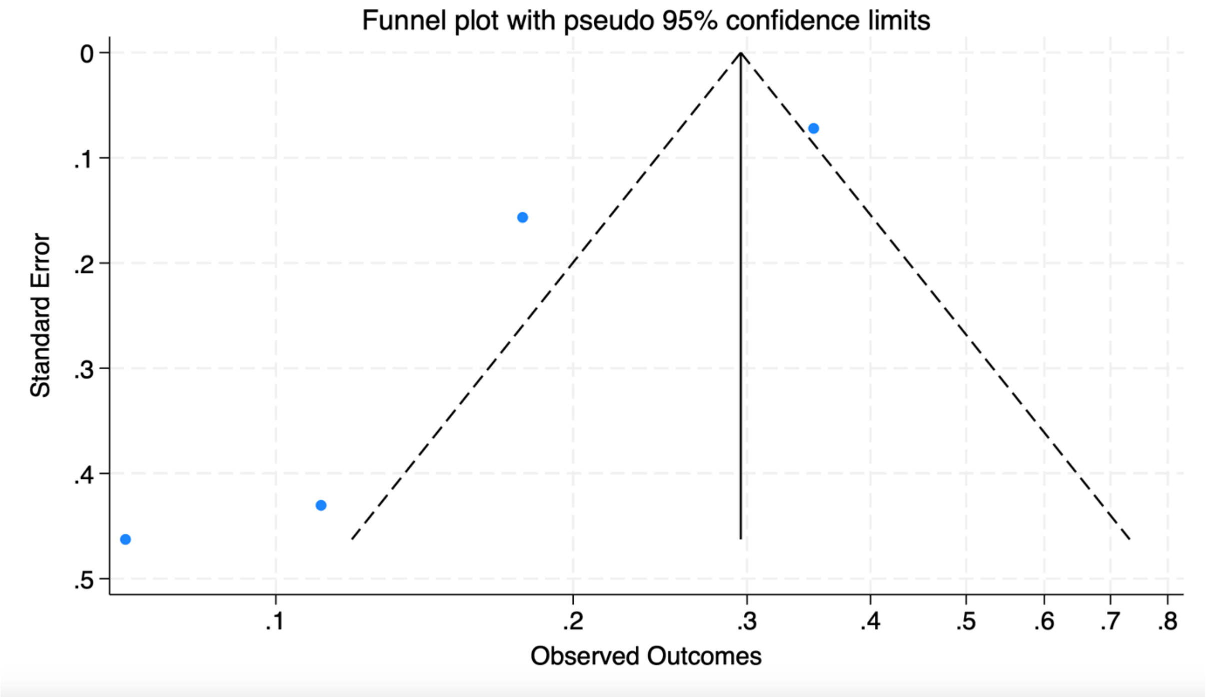

Egger's regression test (p=0.07)

Supplementary Material: Figure 6 - Funnel plot assessment of studies reporting Hazardous Drinking among CHDs

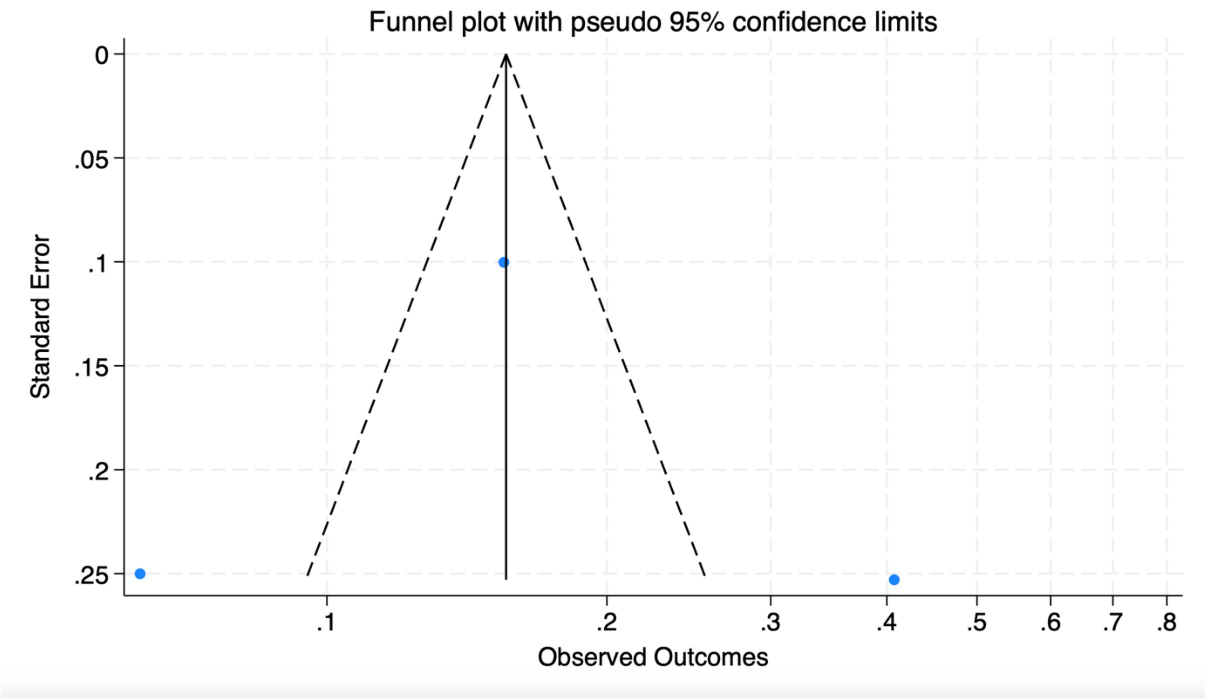

Egger's regression test (p=1.0)
